# Advancements in hydrochlorination of alkenes

**DOI:** 10.3762/bjoc.20.72

**Published:** 2024-04-15

**Authors:** Daniel S Müller

**Affiliations:** 1 Univ. Rennes, CNRS, ISCR (Institut des Sciences Chimiques de Rennes) – UMR 6226, 263 Avenue du Général Leclerc, F-35000 Rennes, France

**Keywords:** addition reactions, alkenes, alkyl chlorides, hydrochlorination, Markovnikov

## Abstract

The hydrochlorination of alkenes has been extensively studied in research and is commonly featured in organic chemistry textbooks as an exemplification of the Markovnikov rule. However, the application of this reaction is typically limited to specific alkenes, such as highly substituted ones, styrenes, or strained systems. Conversely, monosubstituted or 1,2-disubstituted alkenes do not readily react with HCl gas or solutions of HCl gas at practical rates. The challenges associated with hydrochlorination reactions for these "non-activated" alkenes have spurred considerable research efforts over the past 30 years, which constitute the primary focus of this review. The discussion begins with classical polar hydrochlorinations, followed by metal-promoted radical hydrochlorinations, and concludes with a brief overview of recent anti-Markovnikov hydrochlorinations.

## Introduction

The hydrochlorination of alkenes dates back to its discovery by Markovnikov in 1869, who formulated the "Markovnikov rule" as follows: "Experience shows that the halide adds to the least hydrogenated carbon, that is, to the one most susceptible to the influence of other carbon units” [1–2]. In the 1960s and 1970s, various research groups conducted detailed investigations into the kinetics and stereochemistry of hydrochlorination reactions. However, both aspects are highly dependent on the reaction conditions and substrates, and no general conclusions could be drawn [3–9]. Research activity in this field remained relatively dormant until the early 1990s when Kropp's pivotal paper on the surface-mediated hydrochlorination of unactivated alkenes reignited interest [10]. Since then, continuous efforts have been made to enhance the generality, efficiency, and functional group tolerance of hydrochlorination reactions. Recently, several groups reported on metal-catalyzed radical hydrochlorinations [11] and anti-Markovnikov hydrochlorination reactions, highlighting the ongoing challenges in achieving a simple addition of HCl across a simple double bond. During our literature review for this article, we identified two other significant reviews focusing on hydrochlorination reactions. Firstly, an outstanding overview, including extensive research from the former Soviet Union, was reported in 1982 by Sergeev and co-workers [12]. Secondly, the chapter on “*Addition of H-X Reagents to Alkenes and Alkynes*” in comprehensive organic synthesis gives a great overview of hydrochlorinations which were reported between 1940–1980 [13]. Thirdly, Yang and co-workers presented a mini-review on recent hydrochlorinations in 2021 [14]. In this review, hydrochlorination reactions from 1990 to 2023 are comprehensively covered, including several earlier reports to provide a better overall understanding.

**Scheme 1:**
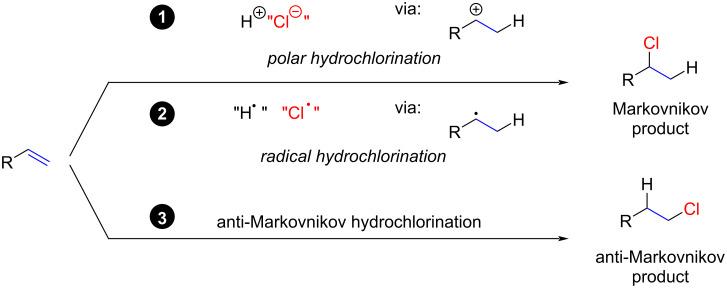
Classes of hydrochlorination reactions discussed in this review.

The hydrochlorination of alkenes can be categorized into three main classes (Scheme 1; only a terminal alkene is shown as a substrate, although polysubstituted and conjugated alkenes can also serve as substrates). 1) Polar reactions: These involve the protonation of the alkene in the first step, providing a carbocation that subsequently reacts with a chloride anion to yield the Markovnikov product. While this ionic mechanism is commonly illustrated in textbooks by showing “naked” cations as intermediates, several recent studies suggest a molecular concerted or simultaneous mechanism [15–19]. 2) Radical hydrochlorinations: These reactions involve the in situ formation of a carbon-centered radical, which is then trapped by an appropriate chlorine source. 3) anti-Markovnikov products: This category describes a new field in hydrochlorination reactions leading to anti-Markovnikov products via several pathways. We have chosen not to present a fourth class of reactions involving either HCl gas or CuCl_2_ and a Pd catalyst, as reported by Alper [20] and Sigman [21], as these reactions are somewhat exotic and are sufficiently discussed in Yang’s review [14].

It is important to note that we are not aware of any catalytic enantioselective hydrochlorination reactions of alkenes. Conjugate additions of HCl to a complex of α,β-unsaturated acids, incorporated in an α-cyclodextrin, which corresponds to a formal hydrochlorination was reported by Tanaka and co-workers [22–23]. Recently, Jacobsen reported asymmetric Prins cyclizations with HCl solutions [24]. Hence, all the described hydrochlorinations are racemic or diastereoselective reactions.

## Review

### Polar hydrochlorination reactions

**Figure 1:**
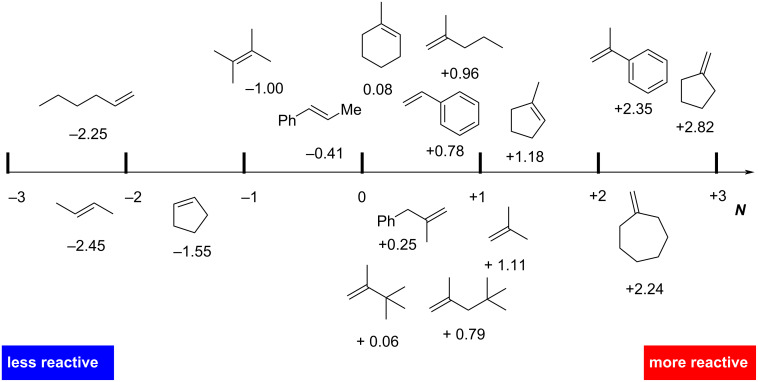
Mayr’s nucleophilicity parameters for several alkenes. References for each compound can be consulted via the database.

To comprehend polar hydrochlorination reactions, a solid understanding of alkene reactivity is essential. Two reactivity scales for alkenes are available in the literature, one considering the reactivity of the alkene itself (Mayr scale) [25] and the other the stability of the corresponding cation after protonation (hydride affinities) [26]. In the polar hydrochlorination reaction, the protonation of the alkene is the rate-determining step. This process can be viewed as the reaction between a nucleophile (alkene) and an electrophile (proton). According to the Mayr–Patz equation log(*k*) = *s*(*N* + *E*), the second order reaction rate constant *k* at 20 °C for a reaction is related to the electrophilicity parameter *E*, the nucleophilicity parameter *N*, and a nucleophile-dependent slope parameters [27]. The nucleophilicity parameter *N*, as proposed by Mayr, provides a dependable estimation of the reactivity of a given nucleophile, such as an alkene in our case (Figure 1). Conveniently, these parameters are freely available on Mayr's database of reactivity parameters [28].

**Figure 2:**
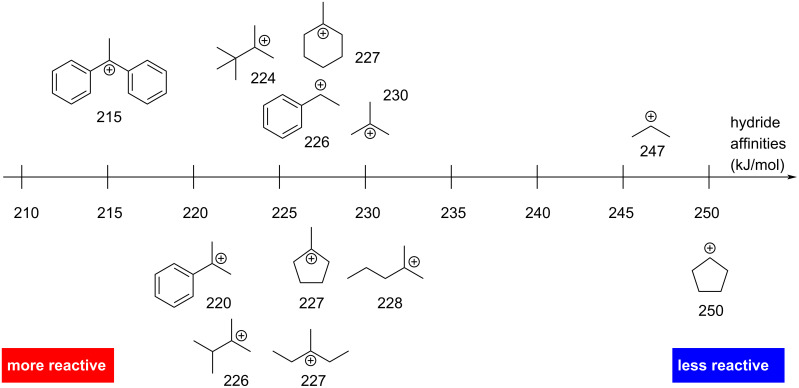
Hydride affinities relating to the reactivity of the corresponding alkene towards hydrochlorination.

On the other hand, one can assess the stability of the in situ-generated cation. The greater its stability, the easier the protonation of the alkene will be, making it more reactive towards hydrochlorination. Thermodynamic and theoretical data provide hydride affinities, which correspond to the negative heat of formation for the combination of a hydride anion with a given cation in the gas phase (Figure 2) [26,29].

It is noteworthy that, in contrast to the hydride affinity scale, the Mayr scale considers energetic differences among alkenes. As demonstrated in the case of methylcyclopentene (Figure 1), the nucleophilicity of the *exo*-double bond is higher compared to the internal double bond. The higher energy of *exo*-alkenes compared to internal alkenes is well known and attributed to a less-effective hyperconjugation of C–H bonds into the alkene π-bond [30].

Before reviewing polar hydrochlorination reactions in detail, it is worth mentioning several statements which were made in the Sergeev review [12]: a) The activation energy for an anti-Markovnikov addition is at least by 30 kJ mol^−1^ higher than for normal addition. Therefore, anti-Markovnikov products are generally not observed. b) In contrast to the reactions with HBr (peroxide effect) [31–32], the formation of *anti*-Markovnikov products is low even in the presence of peroxides or photochemical activation. For instance, Whitmore and co-workers observed only 10–25% of the primary chloride for the reaction of *tert-*butylethylene with HCl in the presence of benzoyl peroxide [33]. c) Several metal halides such as AlCl_3_, SnCl_4_, FeCl_3_, and CuCl exhibit catalytic activities for the hydrochlorination of alkenes. The enthalpy of formation for the hydrogen chloride metal halide complexes are −6 kJ mol^−1^ for SnCl_4_, −8 kJ mol^−1^ for BiCl_3_, −9 kJ mol^−1^ for ZnCl_2_, −15 kJ mol^−1^ for CdCl_2_, −16 kJ mol^−1^ for FeCl_3_, and −41 kJ mol^−1^ for AlCl_3_. d) Addition of chloride-containing salts (e.g., LiCl) accelerate the reaction. e) Traces of water can increase the rate of the reaction.

**Scheme 2:**
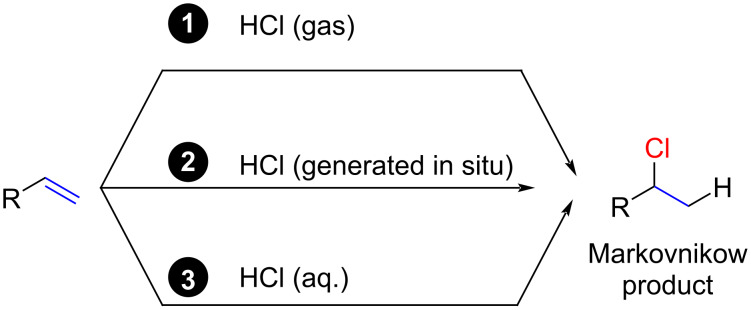
Distinction of polar hydrochlorination reactions.

In light of the numerous research articles on polar hydrochlorination reactions, we have categorized the reports based on the source of HCl (Scheme 2). The first section covers reactions involving HCl gas, typically supplied from an HCl gas cylinder. The second section explores reactions involving the in situ-formation of HCl gas. Lastly, the third section discusses reactions using an aqueous solution of HCl (hydrochloric acid). It is crucial to emphasize the distinction between hydrochloric acid and HCl (gas) or HCl solutions in apolar solvents, as HCl molecules in hydrochloric acid are predominantly dissociated into H_3_O^+^ and Cl^−^. Recent studies by Jacobsen suggest a similar dissociation of HCl for ethereal HCl solutions, which are better described as HEt_2_O^+^ and Cl^−^ [34].

#### Reactions with HCl gas

**Scheme 3:**
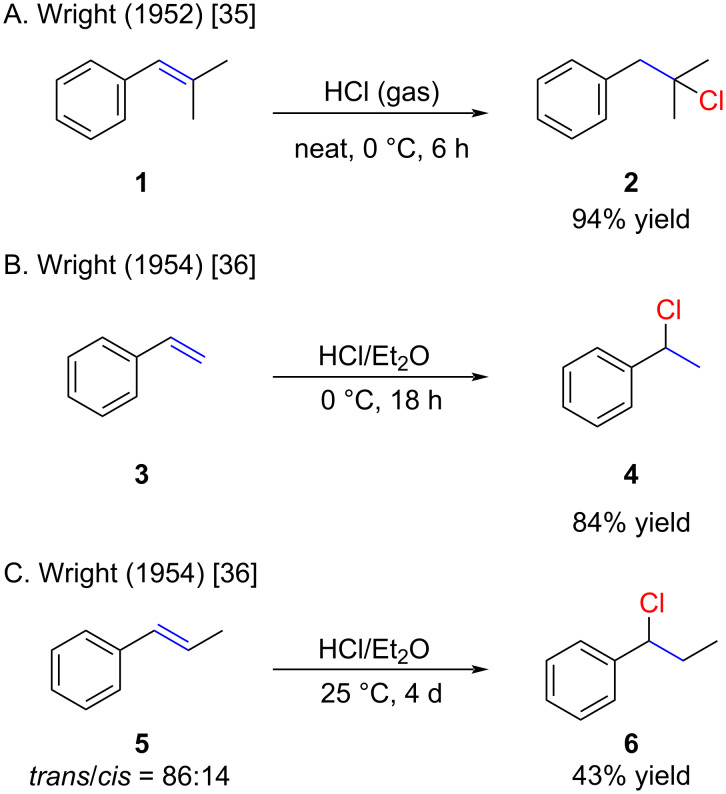
Reactions of styrenes with HCl gas or HCl solutions.

Hydrochlorination reactions with HCl gas were predominant in the field until the 1990s. Generally, HCl gas was bubbled through neat alkene **1** for several hours, as depicted in Scheme 3A [35]. This example highlights an intriguing regioselectivity that might have been challenging to predict through a simple analysis of the stability of the corresponding cations.

Alternatively, the HCl gas was bubbled through a solution of the alkene in diethyl ether at 0 °C or rt (Scheme 3B and 3C) [36]. Despite its effective reaction with styrene (**3**), the reaction displayed sluggish reactivity with 1-propenylbenzene (**5**). It is noteworthy, that the following HCl solutions are commercially available: 4.0 M in dioxane, 3.0 M in methanol, 3.0 M in 1-butanol, 2.0 M in diethyl ether, 3.0 M in cylopentyl methyl ether, 1.0 M in acetic acid.

**Figure 3:**
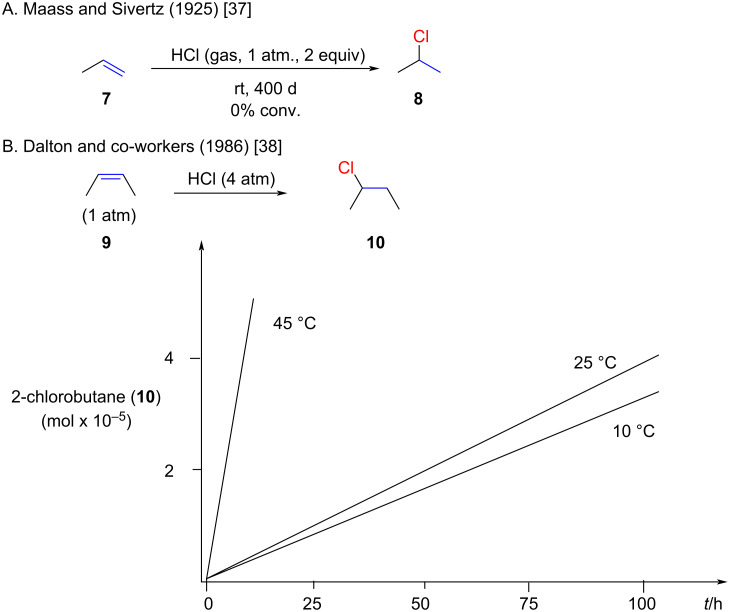
Normal temperature dependence for the hydrochlorination of (*Z*)-but-2-ene.

Terminal aliphatic alkenes, such as prop-1-ene (**7**) do not react with HCl gas at rt and pressures of 1 atm or less (Figure 3A) [37]. In contrast, higher pressures and temperatures significantly accelerate the reaction with aliphatic alkenes (Figure 3B) [38]. A detailed mechanistic analysis for the hydrochlorination with (*Z*)-2-butene (**9**) was carried out by Dalton and co-workers [39]. The reaction between (*Z*)-2-butene (**9**) and hydrogen chloride gas possesses an expected temperature dependence (higher temperature results in higher rates).

**Figure 4:**
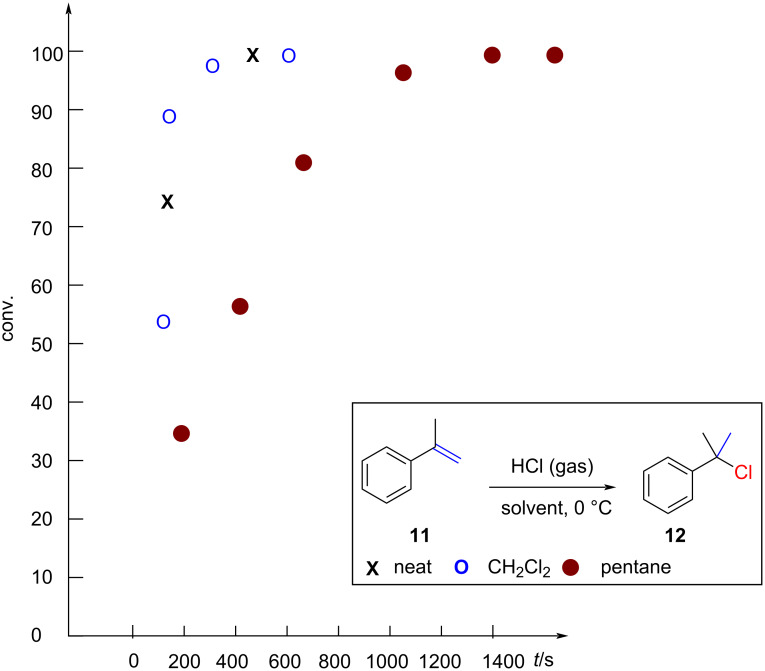
Pentane slows down the hydrochlorination of **11**.

In 1966, Brown and co-worker reported a specialized apparatus enabling the monitoring and control of HCl gas consumption during the reaction [40]. They observed full conversion of α-methylstyrene (**11**) within minutes and suggested that the hydrochlorination is operating within the rate of diffusion control (Figure 4). They also noted that the reaction was significantly slower at room temperature when compared to reactions carried out at 0 °C. However, reaction rates were exclusively reported at 0 and −45 °C, indicating an inverse temperature dependence. Brown also explored the influence of solvents (Figure 4). While reactions conducted in neat α-methylstyrene (**11**) or dichloromethane showed identical kinetics, the reaction was delayed when pentane was employed as a solvent.

**Scheme 4:**
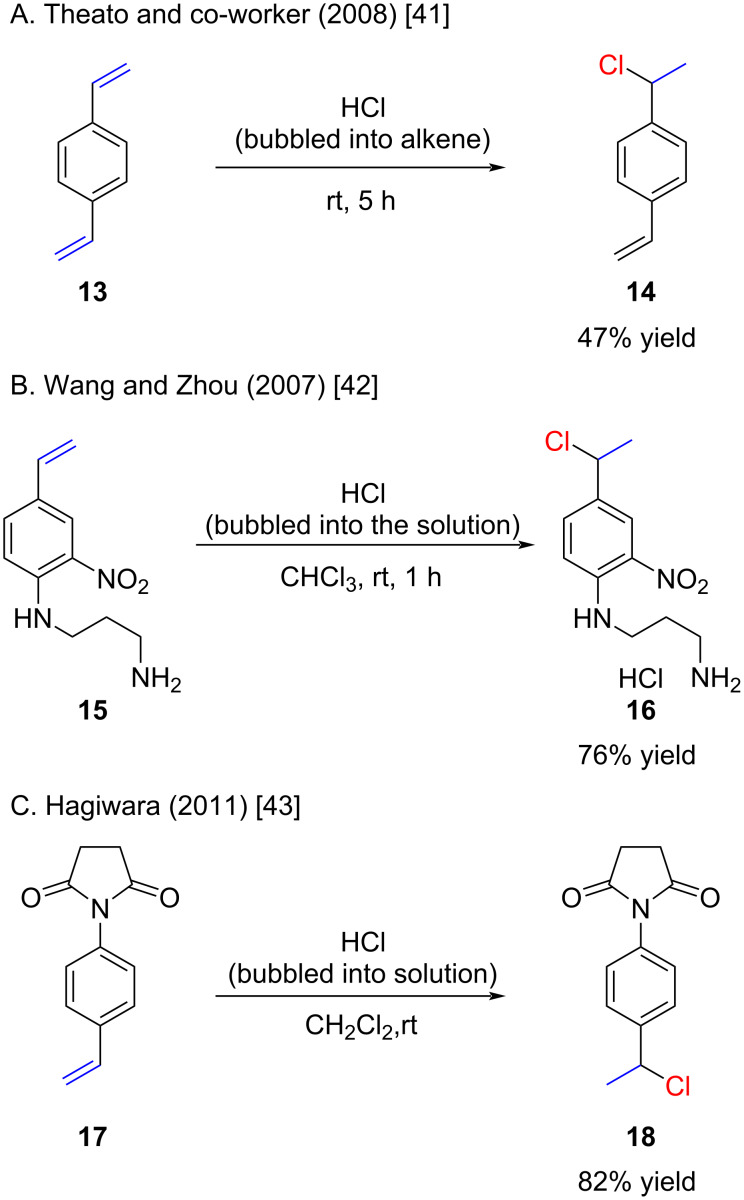
Recently reported hydrochlorinations of styrenes with HCl gas or HCl solutions.

The method of bubbling HCl gas through neat alkenes or solutions of alkenes remains a commonly employed approach, yielding high yields for styrene derivatives (Scheme 4) [41–43]. The example by Theato is remarkable (Scheme 4A), who used HCl (gas) bubbled into neat alkene **13** for 5 hours, and obtained a relatively high yield of the monohydrochlorinated product **14** after distillation [41]. Under these conditions, exclusive formation of the bis-hydrochlorinated product (not shown) might have been anticipated.

**Scheme 5:**
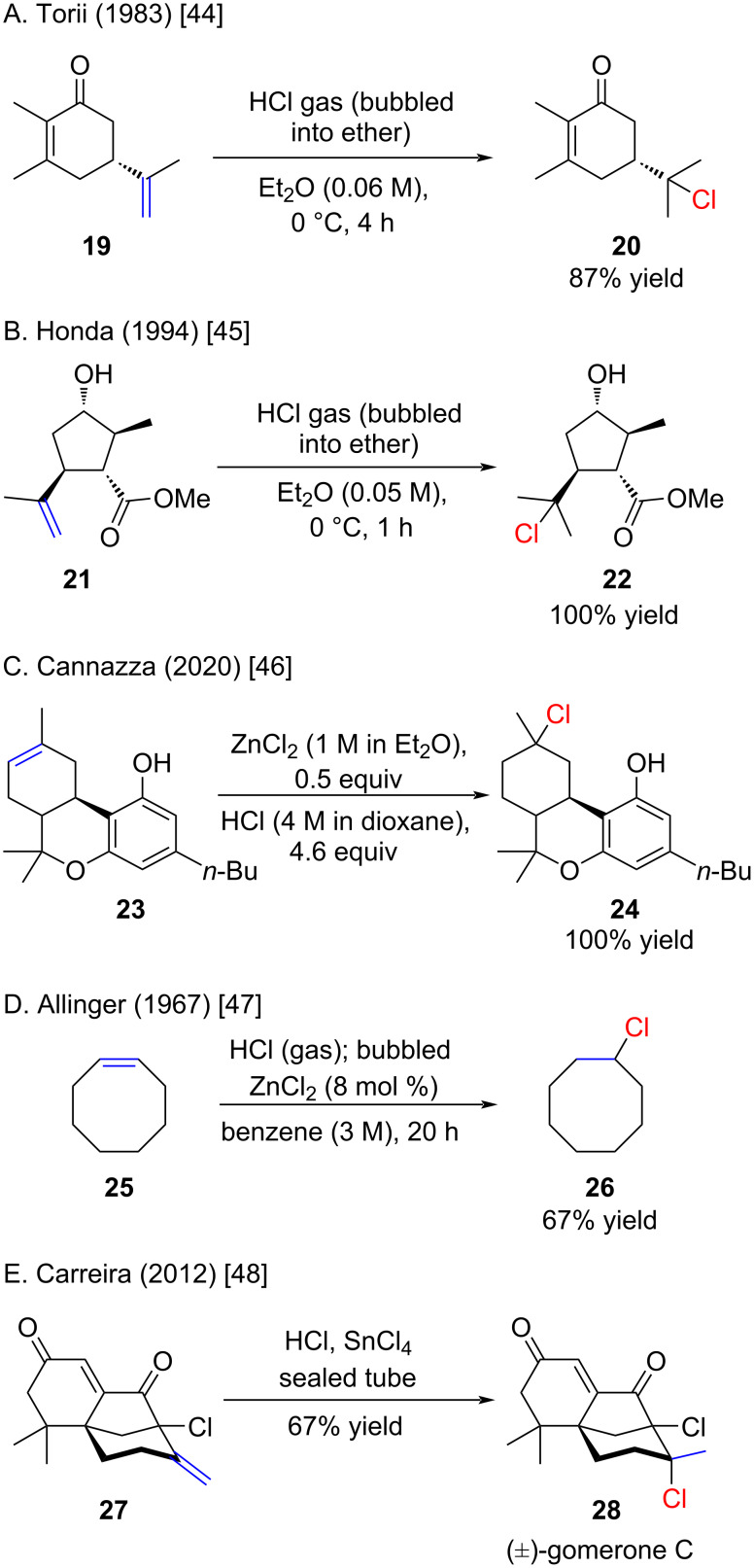
Hydrochlorination reactions with di- and trisubstituted alkenes.

Several examples of the hydrochlorination of more complex molecules were reported (Scheme 5). Torii demonstrated the selective formation of chloride **20** when treating enone **19** with HCl/Et_2_O [44]. This selectivity is notable, especially when compared to reports by other groups indicating the formation of the corresponding phenol derivative under prolonged reaction times (see Scheme 9). Honda reported a quantitative yield in the hydrochlorination of **21** with an ethereal solution of HCl, even in the presence of secondary alcohol and ester functionalities (Scheme 5B) [45]. An application in the synthesis of Δ^9^-tetrahydrocannabutol, the butyl homologue of Δ^9^-tetrahydrocannabinol (Δ^9^-THC), is outlined in Scheme 5C [46]. In this case, ZnCl_2_ was employed as a catalyst, but unfortunately data in the absence of ZnCl_2_ was not provided by the authors. ZnCl_2_ has been previously reported as a catalyst for hydrochlorination reactions, notably in the case of cyclooctene (**25**) with HCl in benzene (Scheme 5D) [47]. The use of ZnCl_2_ as a catalyst for hydrochlorinations dates back to a patent by the British Oxygen Cooperation in 1956 [47]. In 2012, Carreira reported the hydrochlorination of alkene **27**, yielding racemic (±)- gomerone C (**28**) [48].

Grob observed that the stereochemistry of hydrochlorination reactions can be significantly influenced by the solvent or temperature (Table 1) [9,49]. Using liquefied HCl gas, he predominantly obtained *cis*-**30** for the hydrochlorination of 1,2-dimethylcyclohexene (**29**) (Table 1, entry 1), while solutions of HCl gas in ether favored *trans*-**30** (Table 1, entry 2). A similar study, though with lower selectivities, had been conducted by Fahey earlier [8]. In the presence of ammonium salts, a dilute solution of HCl in AcOH resulted in a 7:93 mixture of *cis*-**30** and *trans*-**30** (Table 1, entry 3), whereas an HCl solution in acetyl chloride produced a moderate 73:27 mixture (Table 1, entry 4).

**Table 1:** Stereoselective hydrochlorination of 1,2-dimethylcyclohexene (**29**).

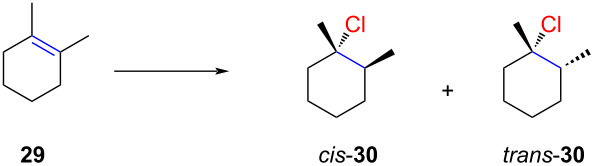			

Entry	Reaction conditions	*cis-* **30**	*trans-* **30**

1	HCl (liquid), −98 °C, 30 min	87	13
2	HCl/Et_2_O, 0 °C, 30 min	5	95
3	0.14 M HCl/AcOH (1.29 equiv), Me_4_NCl (16.6 equiv)	7	93
4	HCl/AcCl, 0 °C, 4 h	73	27

Grob also explored other bicyclic substrates such as octahydronaphthalene (**31**) and hexahydroindene **33** (Table 2 and Table 3). However, in the case of compound **31**, the *cis*-selectivity was relatively low (Table 2, entry 1).

**Table 2:** Stereoselective hydrochlorination of octahydronaphthalene (**31**).

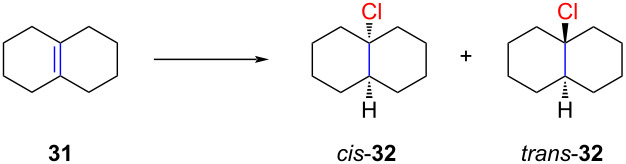			

Entry	Reaction conditions	*cis-* **30**	*trans-* **30**

1	HCl (liquid), −98 °C, 30 min	64	36
2	HCl/Et_2_O, 0 °C, 30 min	2	98

**Table 3:** Stereoselective hydrochlorination of hexahydro-1*H*-indene (**33**).

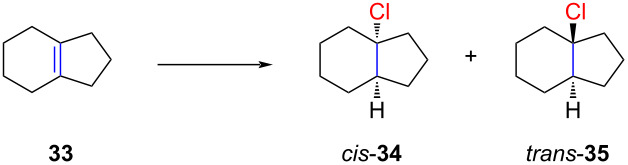			

Entry	Reaction conditions	*cis-* **30**	*trans-* **30**

1	HCl (liquid), −98 °C, 30 min	87	13
2	HCl/Et_2_O, 0 °C, 30 min	7	93

**Scheme 6:**
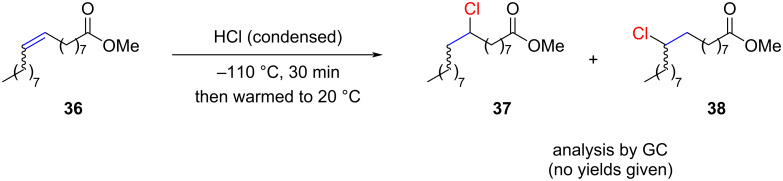
Hydrochlorination of fatty acids with liquified HCl.

Recently, Frøyen and Skramstad studied the hydrochlorination of 1,2-disubstituted alkenes with HCl gas (Scheme 6) [50]. Numerous unsuccessful attempts to hydrochlorinate fatty acids, even with the addition of ZnCl_2_ and LiCl as potential promoters, were reported. Finally, successful reactions conditions were found by using liquefied HCl gas (boiling point of HCl = −85 °C). The researchers concluded that the notable rate acceleration was attributed to the higher concentration of liquid HCl compared to gaseous HCl.

**Scheme 7:**
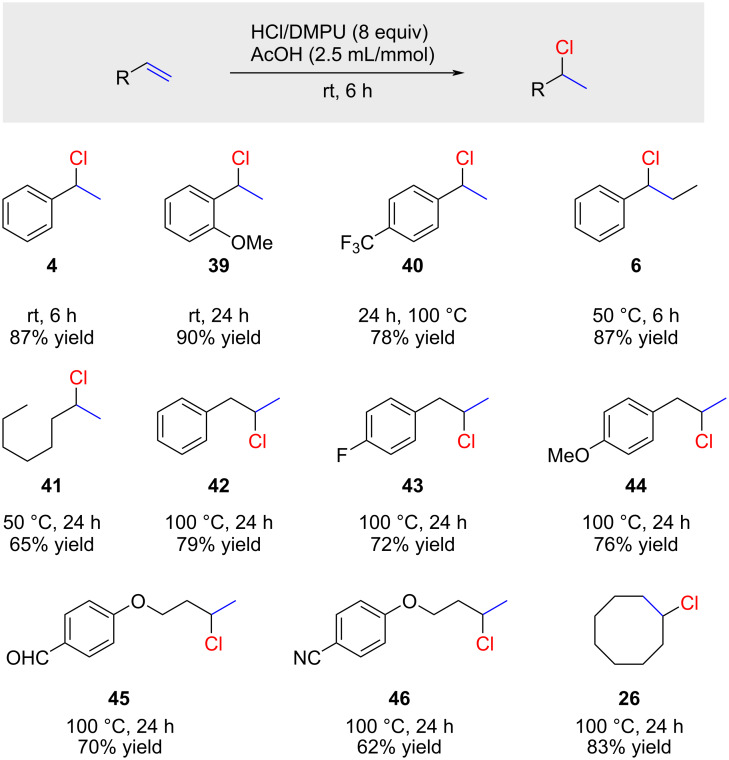
Hydrochlorination with HCl/DMPU solutions.

A systematic study of hydrochlorination reactions with concentrated solutions of HCl gas in DMPU (1,3-dimethyl-3,4,5,6-tetrahydro-2-pyrimidinone) was recently disclosed by Hammond and Xu (Scheme 7) [51]. These solutions were prepared by bubbling HCl gas, generated from NaCl and H_2_SO_4_, into dry DMPU. This yields a 14 M solution of HCl in DMPU, a concentration significantly higher than HCl solutions in other organic solvents. Further enhancement of the reaction was achieved through the addition of acetic acid.

The reaction displays broad generality and tolerates various sensitive functional groups, including aldehyde **45** and nitrile **46**. However, electron-poor styrene, resulting in chloride **40**, or terminal and 1,2-disubstituted alkenes forming chlorides **41**–**46** and cyclooctyl chloride (**26**) necessitated harsher reaction conditions.

As a side note, it should be mentioned that Hutchings and colleagues reported the hydrochlorination of ethylene with Lewis acids on solid supports [52]. However, this work solely focuses on kinetic studies and is therefore not discussed in this report.

#### Reactions with in situ-generated HCl

HCl gas can be generated in situ through the reaction of "reactive" chlorides with a proton donor. For instance, the reaction of acetyl chloride with ethanol is exothermic, accompanied by vigorous HCl gas evolution. It is crucial to emphasize that HCl solutions in MeOH, produced from AcCl and MeOH, pose potential safety hazards, especially in large-scale reactions [53]. Instead of acetyl chlorides, various other reagents, including pivaloyl chloride, oxalyl chloride, SOCl_2_, and TMSCl, can be employed to generate HCl. Numerous proton donors, such as water, alcohol, phenol, or acidic C–H groups, have been reported. In surface-mediated reactions, the proton donor is typically Si–OH or Al–OH. For clarity in discussing subsequent reactions, we have separated the in situ HCl gas synthesis from the hydrochlorination. It is important to note that these reactions are one-pot processes rather than two-pot reactions.

**Scheme 8:**
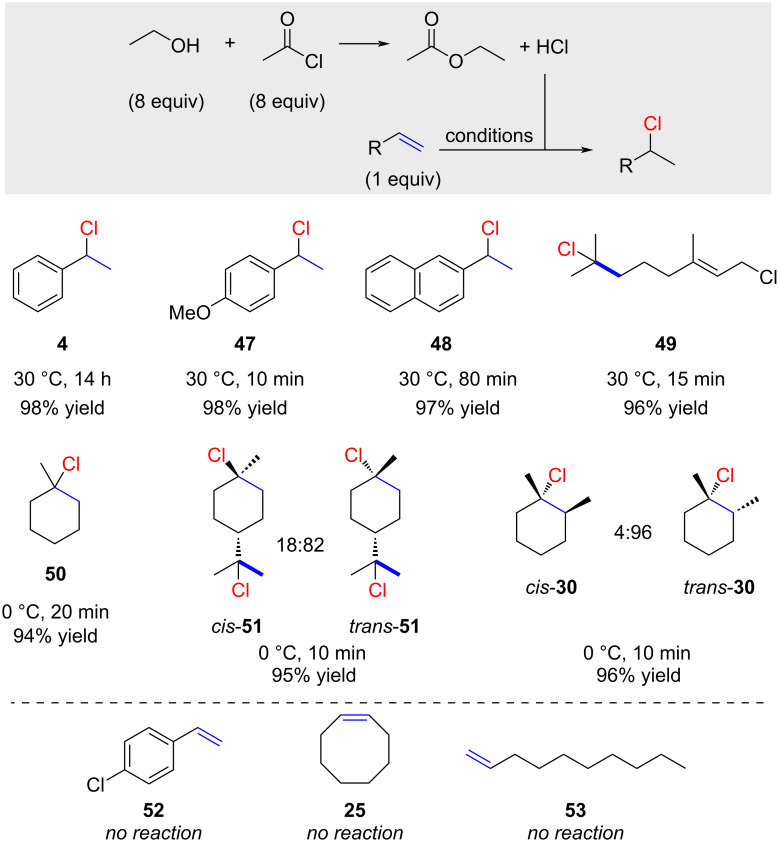
Hydrochlorination with HCl generated from EtOH and AcCl.

Yadav demonstrated that a mixture of 8 equivalents of acetyl chloride with an equimolar amount of ethanol efficiently hydrochlorinates several reactive alkenes (Scheme 8) [54]. Electronic effects are noteworthy; *p*-methoxy-substituted styrene reacted within only 10 minutes to afford chloride **47**, whereas no reaction was observed with *p*-chloro-substituted styrene (product **52**). Geraniol chloride reacted rapidly but only with the more electron-rich double bond (product **49**). 1-Methylcyclohex-1-ene was conveniently transformed into chloride **50** within 20 minutes at 0 °C. Limonene was fully hydrochlorinated affording chloride **51** as a mixture of *cis*- and *trans*-isomers. The hydrochlorination of 1,2-dimethylcyclohexene (**29**) resulted in high selectivity for *trans*-**30**. The authors also showed that an increase in ethanol to 40 equivalents led to a dramatic drop in yield, likely due to an overall lower concentration of HCl. No reaction was observed for terminal and 1,2-disubstituted alkenes such as cyclooctene (**25**) and 1-decene (**53**).

**Scheme 9:**
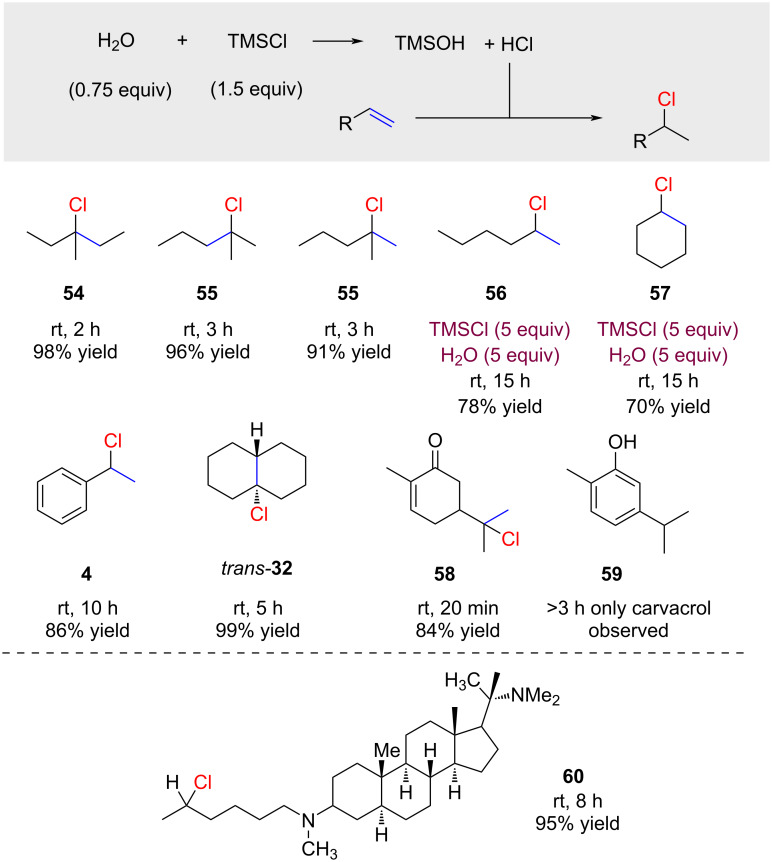
Hydrochlorination with HCl generated from H_2_O and TMSCl.

Boudjouk and co-workers examined PCl_3_, SnCl_4_, SOCl_2_, SiCl_4_, Me_2_SiCl_2_, and Me_3_SiCl as hydrogen chloride sources [55]. They found that PCl_3_ and SnCl_4_ gave the desired hydrochlorination products in acceptable yields but that trimethylchlorosilane (TMSCl) was generally the most useful reagent (Scheme 9). The reactions were typically conducted at room temperature, as elevated temperatures led to a decrease in yield, and lower temperatures were prohibited by the freezing temperature of water. Under slightly more forcing conditions (5 equivalents of TMSCl and 5 equivalents of water), even 1-hexene and cyclohexene reacted successfully at room temperature to afford the corresponding products **56** and **57**. Interestingly, Δ^9,10^-octaline gave exclusively the *trans-*product **32**. The reaction with carvone necessitates careful observation of the reaction time. After 20 minutes, the desired product **58** and only traces of **59** were observed, whereas after 3 h of reaction time **59** was the exclusive product [56]. The method was also recently applied for the synthesis of a derivative of the natural product dictyophlebine (**60**) [57]. Surprisingly, the reported hydrochlorination conditions for the synthesis of **60** differ significantly from the original protocol by Boudjouk (2500 equivalents of H_2_O instead of 1.5 equiv).

A surface-mediated hydrochlorination reaction was reported by Kropp and co-workers [10,58]. They observed that silica gel and alumina, when thermally equilibrated (120 °C, 48 h), facilitated efficient hydrochlorinations when treated with HCl or reactive chlorides. A compelling demonstration of the potent role of silica gel is presented in Table 4. In the absence of silica gel, cycloheptene (**61**), when exposed to a concentrated solution of HCl in dichloromethane, did not show any reaction (Table 4, entry 1). Under the same conditions, in the presence of silica gel, they observed 97% conversion and a GC yield of 62% for chloride **62** (Table 4, entry 2). Further optimization identified thermally treated alumina and SOCl_2_ (2 equiv) as an ideal HCl precursor, affording the product **62** in a 94% yield with 100% conversion in only 18 minutes of reaction time (Table 4, entry 3).

**Table 4:** Hydrochlorination of cycloheptene (**61**).

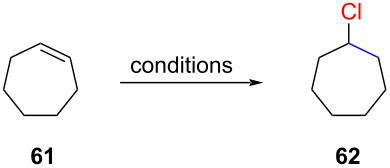		

Entry	Conditions	GC yield of **62** (%)

1	HCl in CH_2_Cl_2_ (sat.), 1 h, −78 °C	0
2	HCl in CH_2_Cl_2_ (sat.), SiO_2_ , 1 h, −78 °C	62
3	SOCl_2_, Al_2_O_3_, rt, 18 min	94

During their investigations, they discovered a correlation between the efficiency of the hydrochlorination reaction and the surface area of the silica gel or alumina. Ethereal solvents were found to yield hydrochlorination reactions only with highly reactive alkenes, such as pinene. Subsequent studies revealed the need to adapt the hydrochlorination procedure for each substrate (e.g., Table 5). For instance, the hydrochlorination of 1-octene (**63**) required a combination of alumina and oxalyl chloride (Table 5, entry 3). It should be noted that this reaction needs to be carried out under a well-ventilated hood due to the evolution of toxic carbon monoxide.

**Table 5:** Hydrochlorination of 1-octene (**63**).

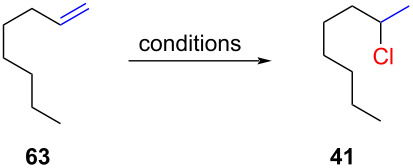		

Entry	Conditions	GC yield of **41** (%)

1	HCl in CH_2_Cl_2_ (sat.), 1 h, −78 °C	0
2	HCl in CH_2_Cl_2_ (sat.), SiO_2_, 1 h, −78 °C	47
3	(COCl)_2_ (2 equiv), Al_2_O_3_, rt, 1 h	97

**Scheme 10:**
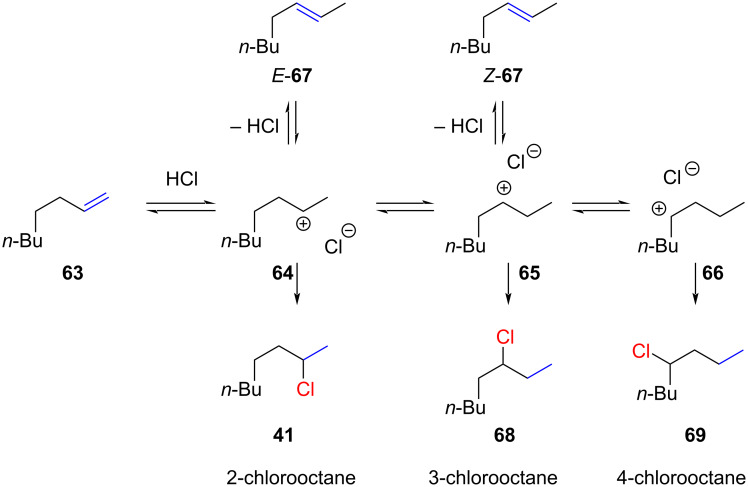
Regioisomeric mixtures of chlorooctanes as a result of hydride shifts.

Kropp and co-workers observed that the remaining 2% of the alkene was a mixture of *E*- and *Z*-octene (**67**) (Scheme 10). They also mentioned in a footnote that 2-chlorooctane (**41**) was contaminated by "some 3-chlorooctane” (**68**). The formation of regioisomers through hydride or alkyl shifts is a common occurrence in hydrochlorination reactions involving secondary cations (Scheme 10).

**Scheme 11:**
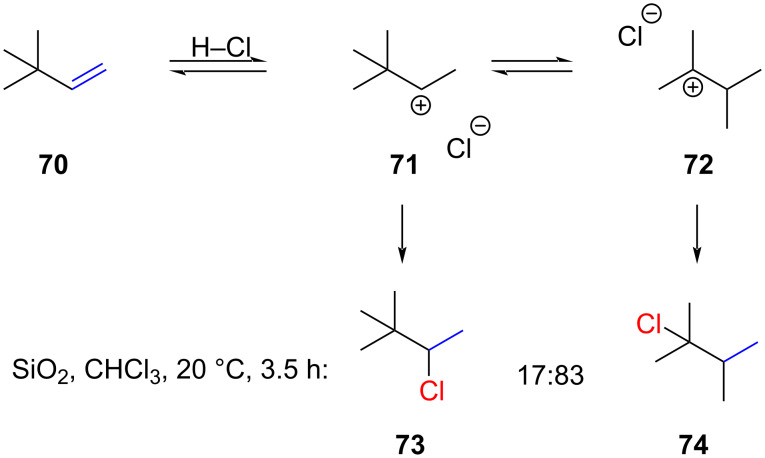
Regioisomeric mixtures of products as a result of methyl shifts.

α-Branched alkenes are particularly prone to alkyl migrations which lead to more stabilized cations (Scheme 11). Thus, the hydrochlorination of *tert*-butylethylene (**70**) produces a mixture of **73** and the rearranged product **74**. The rearrangement of **70** was previously reported by Stevens and Fahey [6,59].

**Scheme 12:**
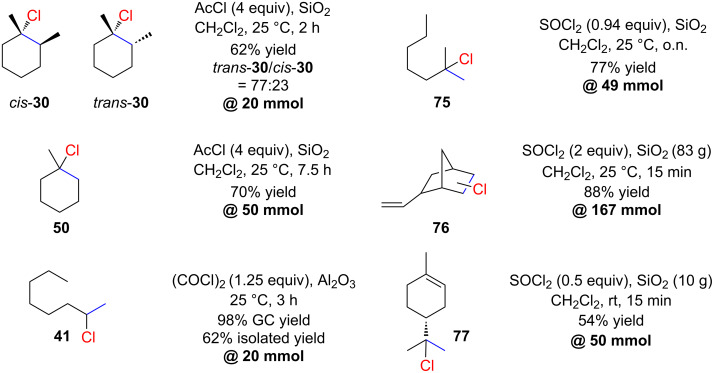
Applications of the Kropp procedure on a preparative scale.

Kropp and co-workers also investigated the stereoselectivity for the hydrochlorination of 1,2-dimethylcyclohexene (**29**) (Scheme 12) [58]. They found a significant dependence of the stereoselectivity on the reaction time. After 1 minute a 88:12 *cis*-**30**/*trans*-**30** ratio was observed which, after 2 hours reaction time, changed to the thermodynamic ratio of 23:77 of *cis*-**30**/*trans*-**30**. The reaction appears to be very robust in terms of scale as illustrated by several examples shown in Scheme 12. More recently, de Mattos applied the Kropp procedure to the delicate monohydrochlorination of limonene on a 50 mmol scale [60]. Little racemization (<7%) of **77** occurred during the reaction. The simplicity of the Kropp protocol resulted in a report in the journal of chemical education [61].

**Scheme 13:**
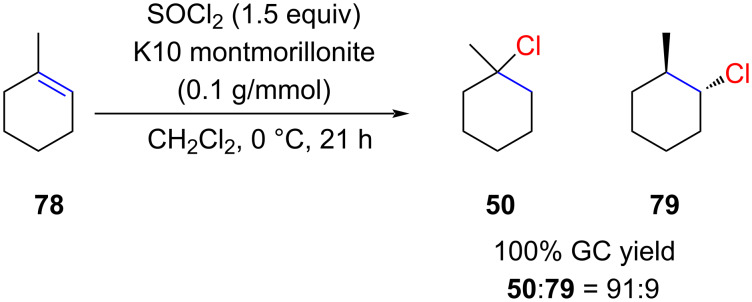
Curious example of polar *anti*-Markovnikov hydrochlorination.

Similar work was reported by Delaude and co-workers [62]. They studied a series of zeolites for the hydrochlorination of alkenes with in situ-generated HCl on a solid support. They found that K10 montmorillonite gave good results for the hydrochlorination of 1-methylcyclohexene (**78**) with SOCl_2_ as the HCl source (Scheme 13). Surprisingly, they not only obtained the expected product **50** but also the regioisomer **79** (anti-Markovnikov product). One plausible explanation for this intriguing observation is that K10 and other zeolites may function both as Brønsted acids and radical initiators [63]. Consequently, it is likely that both ionic and radical pathways are concurrently in operation.

**Scheme 14:**
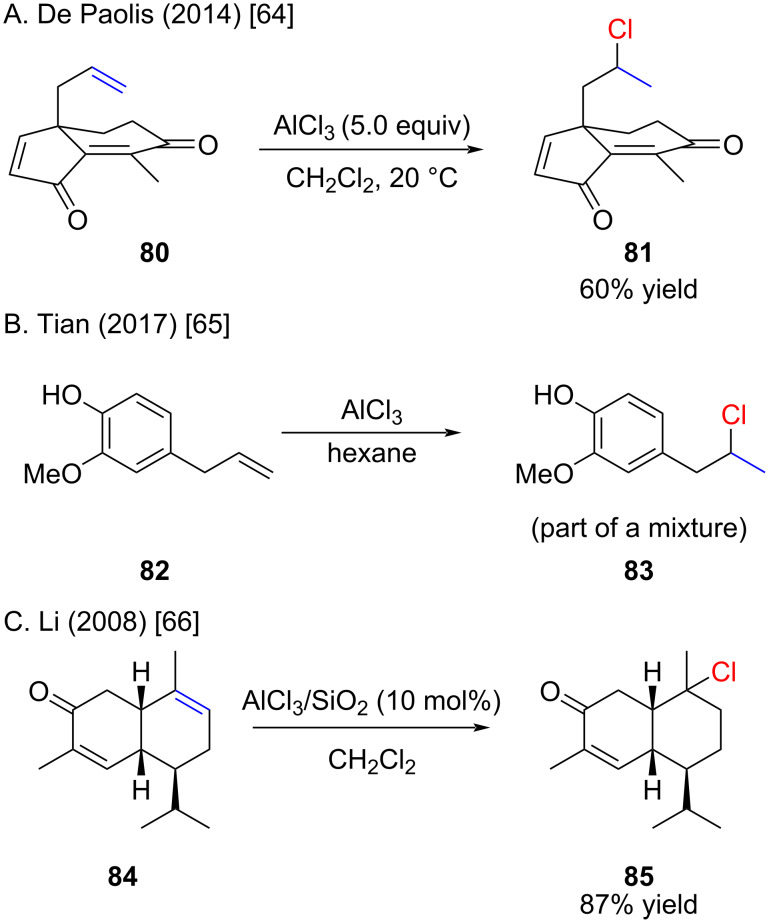
Unexpected and expected hydrochlorinations with AlCl_3_.

The in situ generation of hydrogen chloride with AlCl_3_ and subsequent hydrochlorination reactions were reported in two instances as unexpected products. De Paolis observed the hydrochlorination of a terminal alkene **82** upon treatment with AlCl_3_ (Scheme 14A) [64]. Very likely AlCl_3_ reacted with the acidic enol and gave in situ HCl gas which is responsible for the hydrochlorination. Tian and co-worker reported in a footnote that eugenol (**82**) when treated with AlCl_3_ gives the corresponding hydrochlorination product in a mixture with other products (Scheme 14B) [65]. In this case the reaction of phenol with AlCl_3_ can be suspected as the source of HCl. Another example which lacks experimental details was reported by Li and co-workers (Scheme 14C) [66]. Very likely this reaction was carried out in the presence of HCl gas as a catalyst loading of 10 mol % is certainly not enough to reach 87% yield.

**Figure 5:**
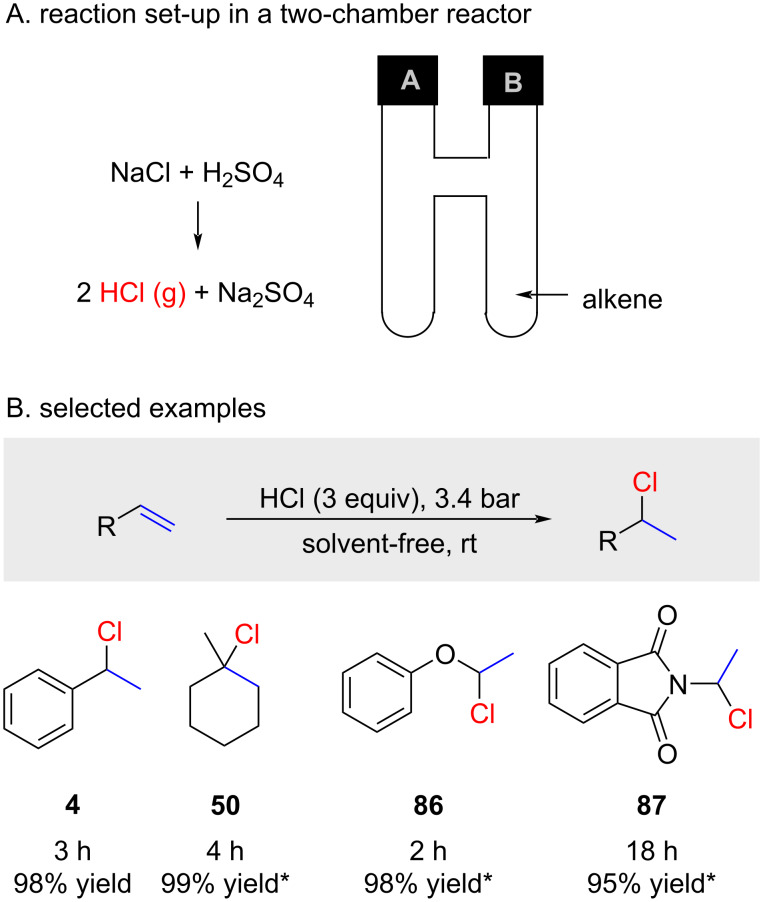
Ex situ-generated HCl gas and in situ application for the hydrochlorination of activated alkenes (* = NMR yield).

HCl gas can also be prepared ex-situ as demonstrated very recently by De Borggraeve and co-workers (Figure 5A) [67]. In a first chamber (A) HCl (gas) was prepared from NaCl and H_2_SO_4_ which then was directed towards a second chamber (B) which contains the alkene under solvent-free conditions. The scope of this reaction (Figure 5B) is limited to reactive alkenes but provides very high yields (yields marked with an asterisk are NMR yields). The addition of DCl was also demonstrated by the use of D_2_SO_4_ instead of H_2_SO_4_.

Not surprisingly, as already discussed in section "Reactions with HCl gas", higher pressures of HCl gas gave more efficient reactions with alkene **88** (Table 6).

**Table 6:** Influence of the HCl gas pressure on the reaction yield.

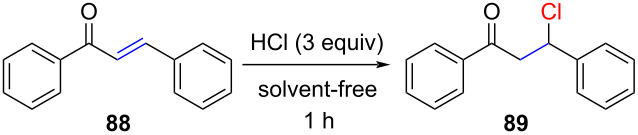		

Entry	*p*HCl (bar)	NMR yield of **87** (%)

1	0.3	7
2	4.8	97

**Scheme 15:**
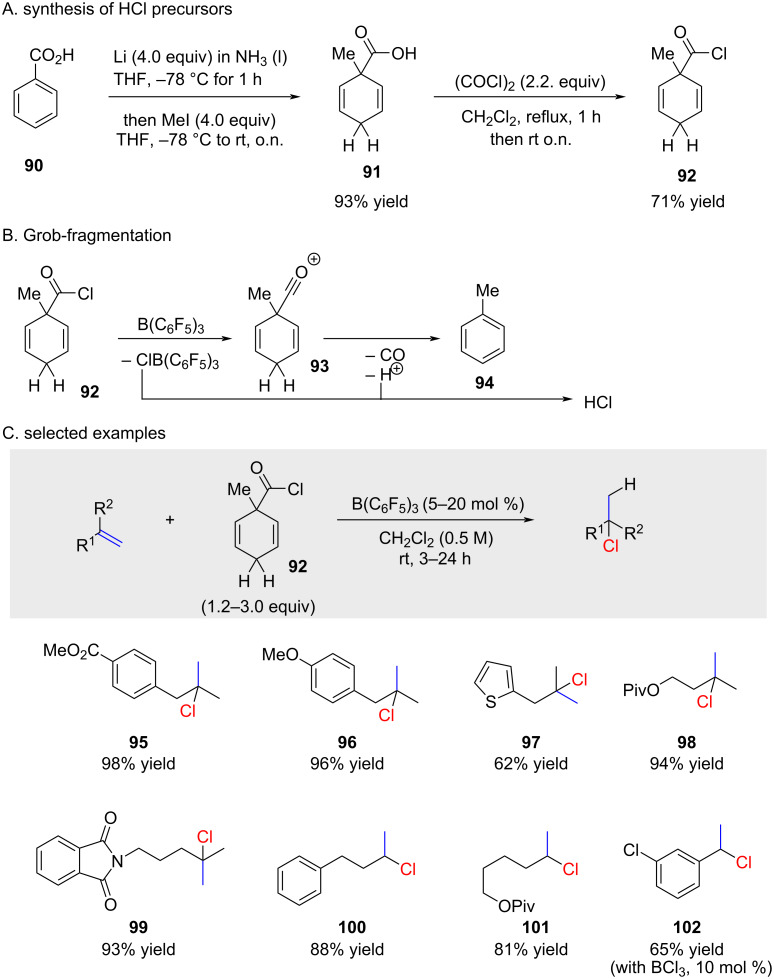
HCl generated by Grob fragmentation of **92**.

In 2022, Oestreich reported the in situ formation of HCl by Lewis acid-induced Grob fragmentation of acid chloride **92** (Scheme 15B) [68]. The inconvenience of this method is that **92** has to be prepared in two steps, including a Birch reduction (Scheme 15A). Activated and non-activated alkenes readily undergo hydrochlorination to form the corresponding tertiary and secondary chlorides (Scheme 15C). However, terminal alkenes required more forcing conditions (3 equiv of **92** and 20% of B(C_6_F_5_)_3_). The oligomerization of 3-chlorostyrene (product **102**) could be prevented when switching to BCl_3_ (10 mol %). Interestingly, toluene (**94**), which is generated as a byproduct of the Grob fragmentation, must react more sluggishly with the generated cationic intermediates compared to chloride, as no alkylations of toluene were reported.

**Scheme 16:**
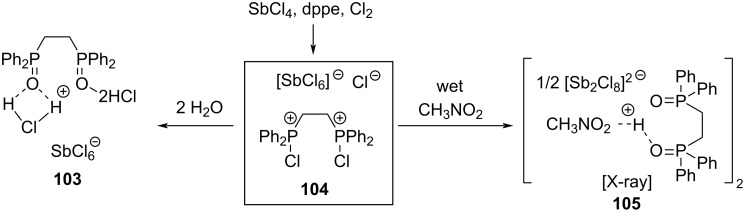
Formation of chlorophosphonium complex **104** and the reaction thereof with H_2_O.

In 2017, the Snyder group published results from their work on hydrochlorination of alkenes in the presence of wet nitromethane and antimony chloride complex **104** which is prepared from dppe (1,2-bis(diphenylphosphino)ethane), chlorine gas, and SbCl_5_ in equimolar ratios (Scheme 16) [69]. They showed that CD_3_NO_2_ did not lead to deuterium incorporation. However, when D_2_O-saturated nitromethane was used >95% D-incorporation took place. Hence the small (500–2000 ppm) content of water in commercially available nitromethane was at the origin of the hydrochlorination reaction (Scheme 16). As a result, the reaction of **104** with water forms a dissociated hydrogen chloride aggregate in the form of complexes **103** or **105**. An X-ray structure of complex **105** was reported and the reaction **104** → **105** is described in the report by Snyder, though not stoichiometric balanced. These complexes seem to play a pivotal role in the hydrochlorination reaction. To confirm that the reaction was not solely a result of an HCl solution in nitromethane, they tested the hydrochlorination of alkene **106** with HCl in acetic acid in the presence of dry nitromethane (entry 1 in Table 7). However, it would have been more interesting to replicate entry 1 using moist CH_3_NO_2_, considering the well-established knowledge that water can significantly accelerate hydrochlorination reactions [70–71]. The data suggests that just a minimum amount of water is necessary to hydrolyze the initial complex as excess water slows down the reaction with complex **105** (compare entries 2 and 3 in Table 7). The reaction with only oxidized ligand (dppeO_2_) also works indicating that structure **103** could also be the active hydrochlorination complex (compare entries 4 and 5 in Table 7).

**Table 7:** Control reactions for the hydrochlorination reaction of alkene **106**.

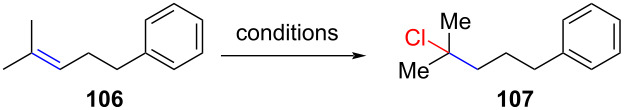			

Entry	conditions	Time (h)	Yield **108** (%)

1	1.0 M HCl in EtOAc, dry CH_3_NO_2_	40	23
2	complex **105** (2.2 equiv), 1.0 M HCl in EtOAc, dry CH_3_NO_2_	16	68
3	complex **105** (2.2 equiv), 1.0 M HCl in EtOAc, wet CH_3_NO_2_	40	23
4	dppeO_2_, 1.0 M HCl in EtOAc, dry CH_3_NO_2_	16	64
5	complex **104** (2.2 equiv), wet CH_3_NO_2_	16	79

**Scheme 17:**
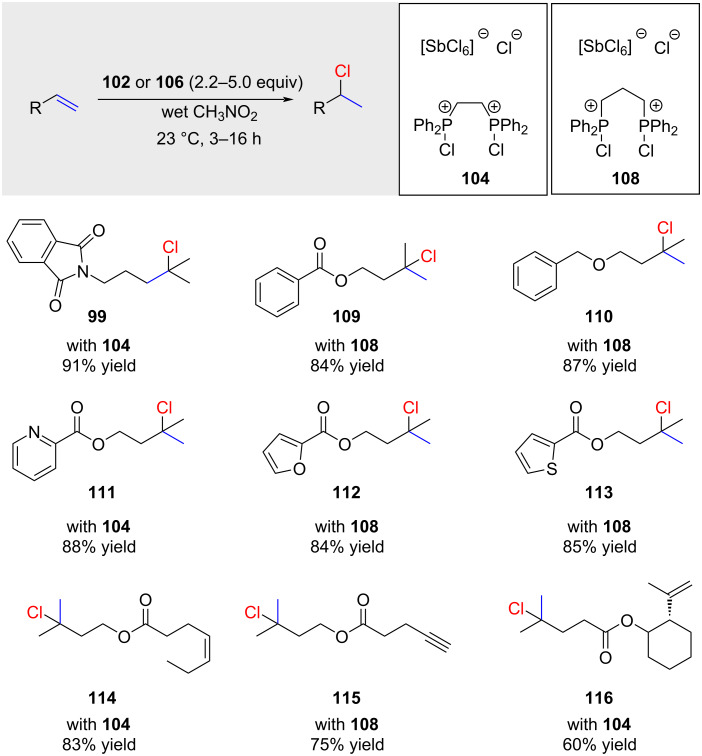
Snyder’s hydrochlorination with stoichiometric amounts of complex **104** or **108**.

The applicability of the reaction is confined to highly reactive 1,1-disubstituted or trisubstituted alkenes (Scheme 17). Remarkably, various functional groups were well tolerated, and the product **114** was obtained with unexpectedly high regioselectivity.

In 2022, Paquin and colleagues devised a practical hydrochlorination reaction, utilizing a mixture of methanesulfonic acid and hand-ground CaCl_2_ in acetic acid as a mild hydrochlorination reagent (Table 8) [72]. Notably, the addition of acetic acid substantially enhanced the yield of chloride **109** (Table 8, entry 3).

**Table 8:** Optimization reactions for the hydrochlorination reaction with CaCl_2_.

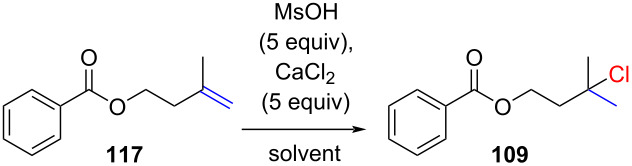		

Entry	Reaction conditions	NMR yield of **109** (%)

1	CaCl_2_ beads (CH_2_Cl_2_)	21
2	CaCl_2_ hand-ground (CH_2_Cl_2_)	61
3	CaCl_2_ hand-ground and AcOH (5 mL/mmol)	90

**Scheme 18:**
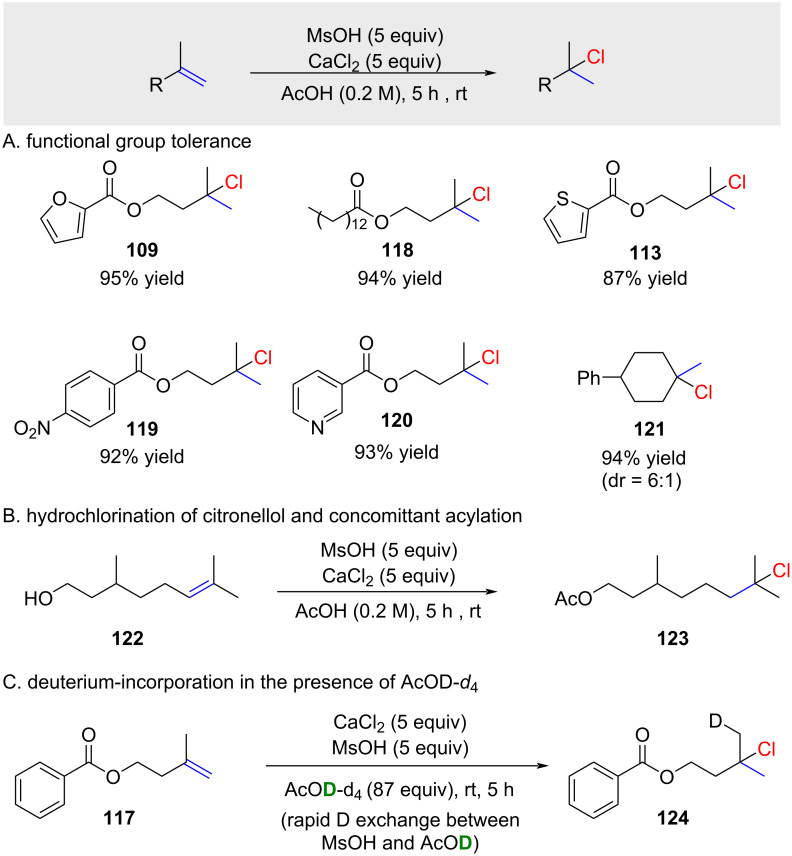
In situ generation of HCl by mixing of MsOH with CaCl_2_.

The functional group tolerance of the reaction appears to be similar to the one reported by Snyder [69] (compare Scheme 17 and Scheme 18A). The reaction worked exclusively for reactive alkenes such as 1,1-disubstituted or trisubstituted alkenes. No conversion was observed for terminal alkenes and 1,2-substituted alkenes. When exposing citronellol (**122**) to the reaction conditions, the alcohol was rapidly converted to the acetate and the alkene to the corresponding chloride **123** (Scheme 18B). A deuterium label experiment demonstrated the rapid H/D exchange with the deuterated solvent (Scheme 18C). This reaction is synthetically interesting due to the significantly lower cost of AcOD-*d*_4_ compared to deuterium-enriched methanesulfonic acid.

#### Hydrochlorination with hydrochloric acid

**Scheme 19:**
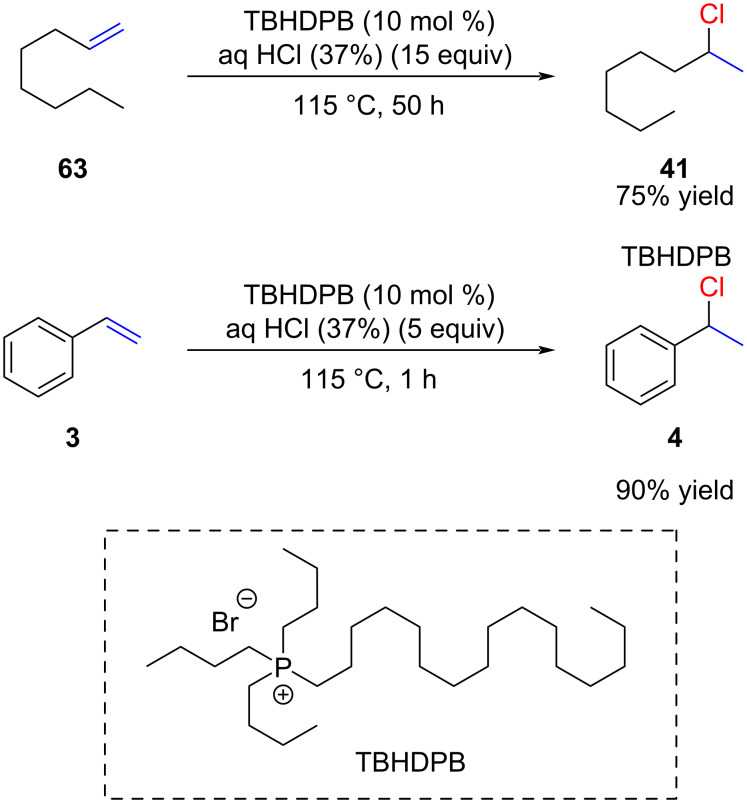
First hydrochlorination of alkenes using hydrochloric acid.

The application of 37% hydrochloric acid for alkene hydrochlorination is a surprisingly recent development. Remarkably, Rolla, in 1980, was the pioneer in reporting hydrochlorination with hydrochloric acid 110 years after Markovnikov's initial hydrochlorination report [73]. Although seemingly unbelievable, the reasons for this delayed progress will become apparent in the subsequent discussion. Rolla introduced a noteworthy enhancement by incorporating 10 mol % of tributylhexadecylphosphonium bromide (TBHDPB; CAS: 14937-45-2) as a phase-transfer catalyst, enabling the hydrochlorination of 1-octene (**63**) in the presence of 15 equivalents of hydrochloric acid (Scheme 19). The reaction necessitates heating to 115 °C for 2 days to achieve 90% conversion and a 75% isolated yield of **41**. Conversely, the reaction with styrene (**3**) is complete within one hour, yielding **4** with 90% yield and 99% conversion. A drawback of the Rolla protocol is the cost associated with the phase-transfer catalyst, and that the crude mixture requires purification through distillation or column chromatography. Another inconvenience is the high reaction temperature which limits the functional group tolerance.

**Scheme 20:**
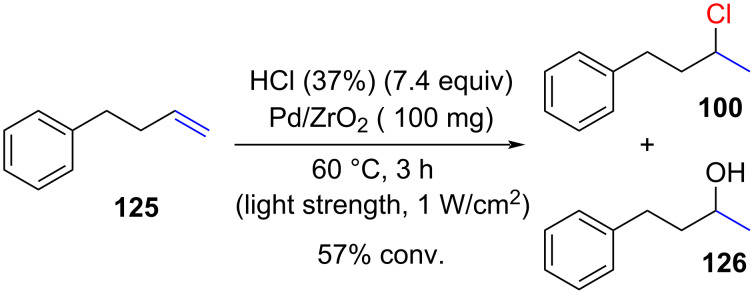
Visible-light-promoted hydrochlorination.

Yang reported a hydrochlorination promoted by visible light over platinum, gold, and palladium supported on zirconia [74]. The reaction demonstrated moderate efficiency, yielding an 85:15 mixture of products **100** and **126** with low conversion (Scheme 20).

**Scheme 21:**
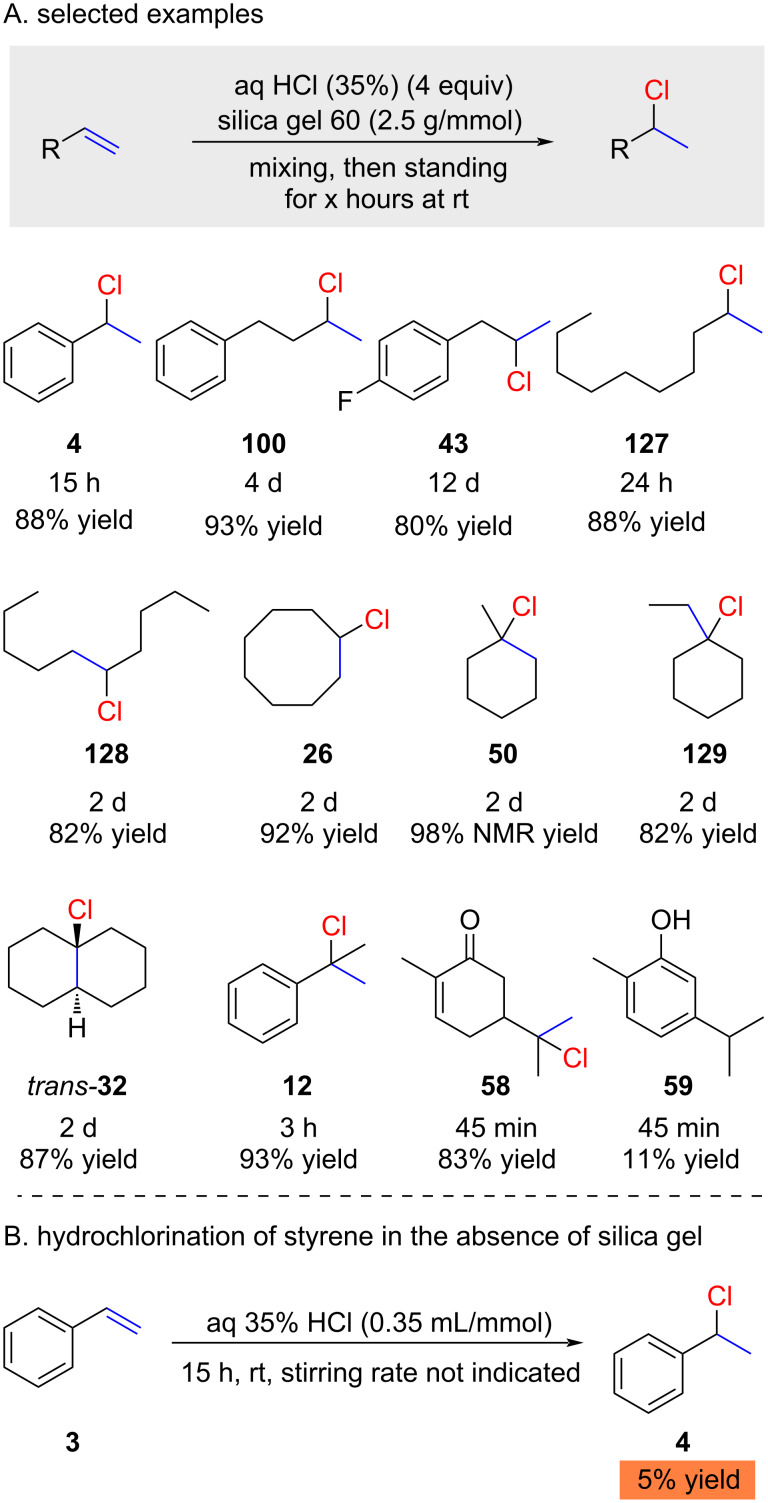
Silica gel-promoted hydrochlorination of alkenes with hydrochloric acid.

The use of hydrochloric acid in combination with silica gel was very recently reported by Tanemura (Scheme 21A) [75]. The authors proposed that water in hydrochloric acid is absorbed by silica gel, producing a system similar to the one previously reported by Kropp (HCl adsorbed on the silica gel surface; e.g., Table 4). To effectively absorb the water content of 35% hydrochloric acid, 625 mg of silica gel are required per mmol of HCl. HCl absorbed on hydrated silica gel has proven to be efficient for various alkenes, including non-activated terminal alkenes. The yields are generally high, but the reactions are somewhat slow (up to 12 days reaction time; product **43**). It is unclear if these long reaction times are optimized or not. For example, the highly reactive 1-methylcyclohexene (**78**) requires 2 days (product **50**), whereas the less reactive styrene (**3**) only requires 15 hours to give high yields for product **4**. It is noteworthy that Tanemura demonstrated that hydrochloric acid in the absence of silica gel gives only very sluggish reactions (Scheme 21B). This is probably the reason why hydrochloric acid was ignored as a reagent for hydrochlorination reactions for more than a century.

Most recently, our group revisited the hydrochlorination reaction and optimized the stoichiometry and the stirring rate for the hydrochlorination of alkenes with fuming hydrochloric acid (37%) (Table 9) [76]. We found that for biphasic mixtures high stirring rates (1500 rpm) gave significantly improved conversions and that 1 mL of fuming 37% hydrochloric acid per mmol of substrate gave the best results. Under these conditions the hydrochlorination of 1-methylcyclohexene (**78**) gave 81% GC yield in only 20 minutes (Table 9, entry 3). The addition of acetic acid (1.0 mL/mmol) further improved the reaction rates and afforded a 98% GC yield (entry 4 in Table 9).

**Table 9:** Optimization reactions.^a^

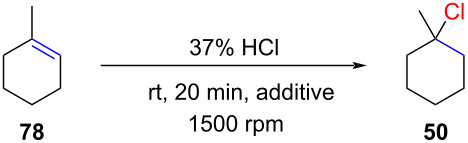			

Entry	HCl (mL/mmol)	Additive (mL/mmol)	GC yield (**50**) (%)

1	0.1	none	11
2	0.5	none	61
3	1.0	none	81
4	1.0	AcOH (1.0 mL)	98

^a^rpm = revolutions per minute.

**Scheme 22:**
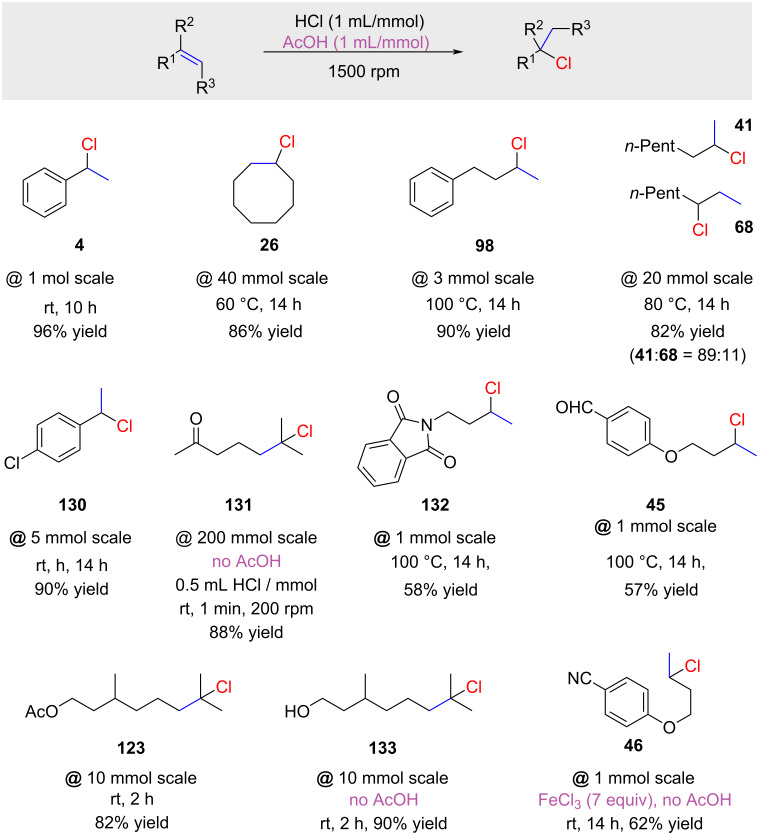
Hydrochlorination with hydrochloric acid promoted by acetic acid or iron trichloride.

The methodology was applied to a significant number of substrates with many reactions being carried out on a ≫1 mmol scale (Scheme 22). The hydrochlorination of styrene (**3**) was even carried out on a one-mole scale demonstrating the robustness of this procedure (product **4**). Not unexpectedly, the conditions for the hydrochlorination of alkenes had to be adapted for each alkene. Whereas reactive alkenes gave the corresponding chlorides within several hours at room temperature, terminal alkenes required harsher reaction conditions. 1-Octene (**63**) gave under these conditions a mixture of the regioisomers **41** and **68**. The formation thereof was previously discussed in Scheme 10. Reactions with polar substrates such as 6-methylhept-5-en-2-one resulted in homogenous reaction conditions and did not require vigorous stirring (product **128**). As previously observed by Paquin and co-workers citronellol gave the corresponding acetate **123** when exposed to hydrochloric acid in the presence of acetic acid. Omission of acetic acid let to alcohol **133**. Screening of various additives identified that hydrochloric acid saturated with ZnCl_2_ or FeCl_3_ significantly improved the reaction rate. FeCl_3_-saturated solutions allowed carrying out the hydrochlorination of terminal aliphatic alkenes at room temperature. Under these conditions even a nitrile group could be preserved (**46**). The FeCl_3_-promoted hydrochlorination with HCl gas was previously reported by Mayo and Scher [31,77]. This methodology is of great practical interest as the starting materials are inexpensive bulk chemicals and the reactions can be carried out under air without any particular precautions.

### Radical hydrochlorination reactions

**Figure 6:**
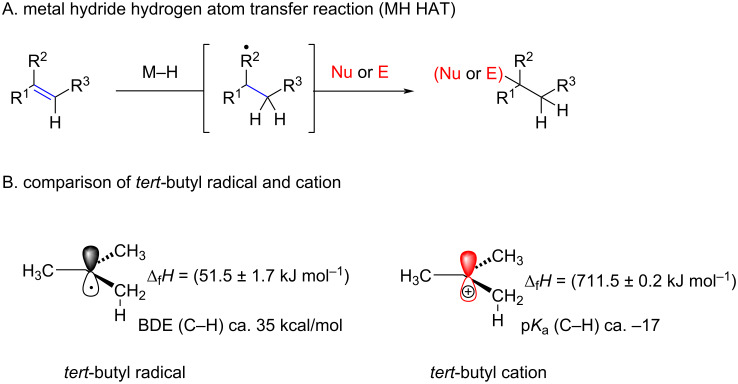
Metal hydride hydrogen atom transfer reactions vs cationic reactions; BDE (bond-dissociation energy).

**Figure 7:**
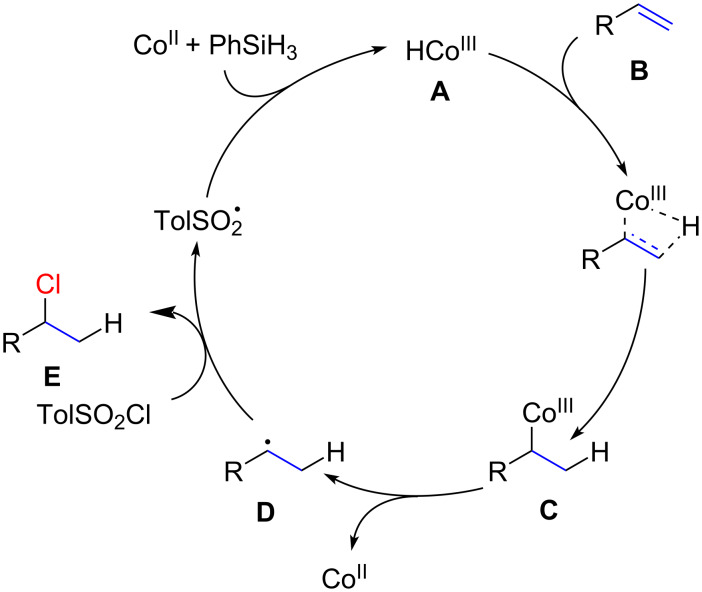
Mechanism for the cobalt hydride hydrogen atom transfer reaction reported by Carreira.

Cobalt and iron-promoted radical hydrochlorination reactions are part of the large family of metal hydride hydrogen atom transfer (MH HAT) reactions (Figure 6A). As pointed out by Shenvi in a recent review [11], the major difference between traditional polar hydrochlorinations of alkenes and MH HAT is that the latter is far more chemoselective and proceeds under “milder” conditions. As shown in Figure 7B, carbocations or carbenium ions are highly energetic species which tend to react unselectively according to the reactivity–selectivity principle. In contrast, MH HAT produces relatively stable radicals which is demonstrated by, e.g., the strong difference of heat of formation of the *tert*-butyl radical and cation (Figure 7B) [78]. Another advantage of the MH HAT process is that the α-C–H bond in the corresponding radical is comparatively stable, whereas a carbocation has superacidic α-C–H bonds with a p*K*_a_ of ≈ −17 [79]. Therefore, polar hydrochlorination reactions are in competition with elimination reactions which is not the case for radical reactions.

According to these differences it is clear that applications of metal-promoted radical hydrochlorination reactions will mainly focus on complex molecules and substrates where classical hydrochlorination reactions fail.

**Scheme 23:**
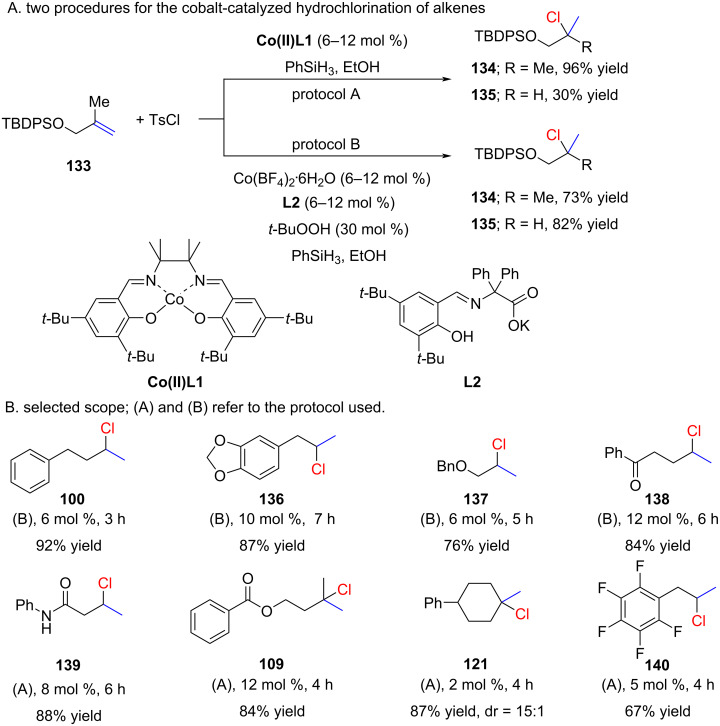
Carreira’s first report on radical hydrochlorinations of alkenes.

Carreira and Gaspar were the pioneers in reporting the metal-catalyzed hydrochlorination of alkenes based on MH HAT reactions (Scheme 23) [80]. They discovered that a combination of a cobalt catalyst, a silane, and tosyl chloride promoted the hydrochlorination of terminal unactivated alkenes. The scope of the reaction is relatively broad when employing two protocols (Scheme 23A). Generally, protocol A works well for the synthesis of tertiary chlorides, whereas protocol B is preferable for the synthesis of secondary chlorides (Scheme 23B). Surprisingly, free alcohols required protection even though the reaction is carried out in ethanol as a solvent (e.g., alkene **133**). Another unexpected finding was that styrenes, previously described by Carreira in hydrohydrazination [81] and hydrocyanation reactions [82], failed to undergo the hydrochlorination reaction. Notably, the synthesis of compound **100** was reported on a 50 mmol scale in Organic Syntheses [83].

The proposed catalytic cycle is shown in Figure 7 and involves the following steps. First, a cobalt hydride complex **A** is formed in situ from Co(II) complex and the silane. Then, regioselective alkene hydrocobaltation takes place. This step is highly regioselective, placing the cobalt atom on the higher-substituted carbon atom to furnish intermediate species **C**. The irreversibility of the hydride addition and the regioselectivity thereof were supported by a deuterium labelling study with PhSiD_3_. The next steps involve homolytic cleavage of the cobalt–carbon bond to yield a carbon-centered radical **D** which is then trapped by TsCl to produce the corresponding alkyl chloride **E**.

Detailed mechanistic studies on a related reaction were recently reported by Shenvi and co-workes [84]. These studies alternatively suggest that intermediate **C** could also be the result not of a non-simultaneous addition of the Co–H to the alkene but of a step-wise radical addition.

**Scheme 24:**
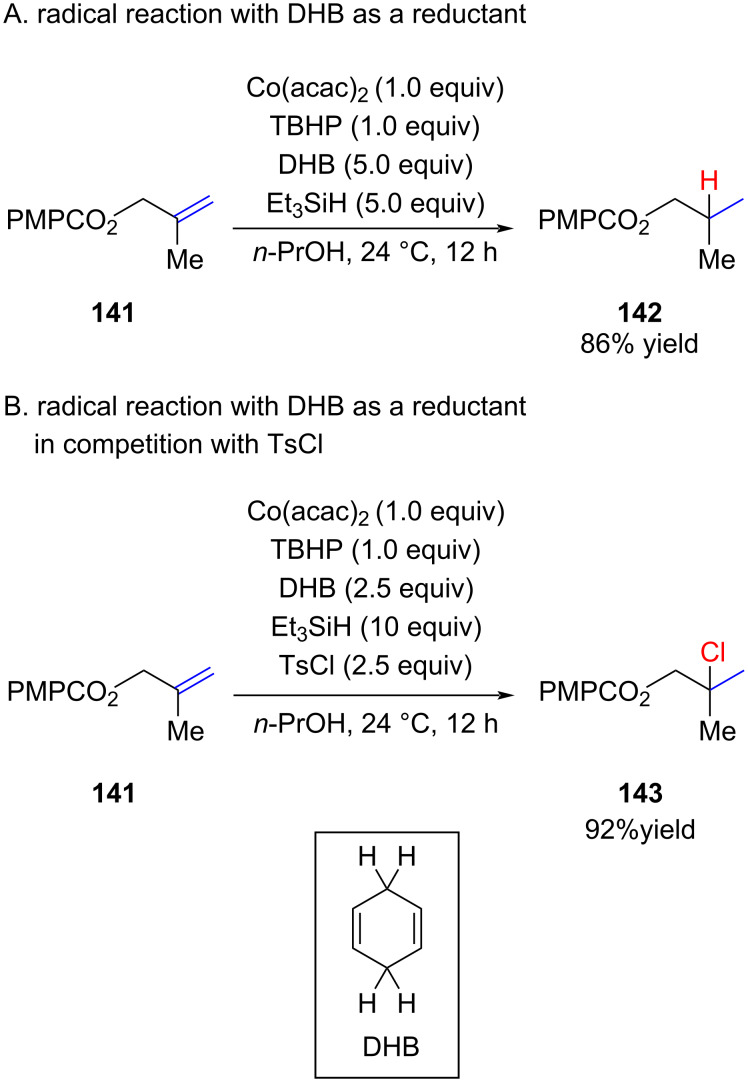
Radical “hydrogenation” of alkenes; competing chlorination reactions.

A similar procedure was reported by Herzon [85]. His study focused on the use of two reductants, triethylsilane and 1,4-dihydrobenzene (DHB) (Scheme 24). He showed that in the presence of DHB, the intermediate radical could be trapped by DHB as a hydride donor and thus give the fully reduced product **142**. However, when both DHB and TosCl were present, the reaction of the radical with TosCl was significantly faster leading to **143** in 92% yield.

**Scheme 25:**
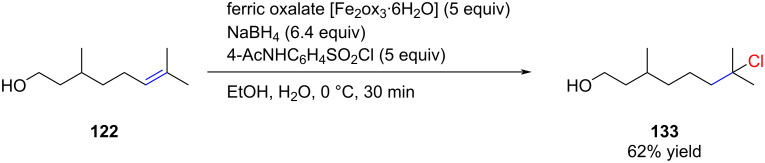
Bogers iron-catalyzed radical hydrochlorination.

In 2012, Boger demonstrated the efficiency of iron(III) catalysts for the hydrochlorination of activated alkenes [86]. Subjecting citronellol (**122**) to the optimized reaction conditions resulted in the formation of chloride **133** with a yield of 62% (Scheme 25). It is worth noting that the iron-catalyzed procedure tolerates free alcohols, a distinction from Carreira's protocol [80].

**Scheme 26:**
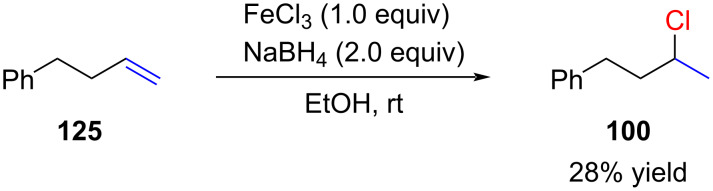
Hydrochlorination instead of hydrogenation product.

In 2014, the Thomas group reported on the formal hydrogenation of alkenes with Fe(OTf)_3_ in the presence of NaBH_4_ [87]. During their studies they noted that FeCl_3_ was able to perform hydrochlorination reactions with alkenes albeit in low yields (Scheme 26).

**Scheme 27:**
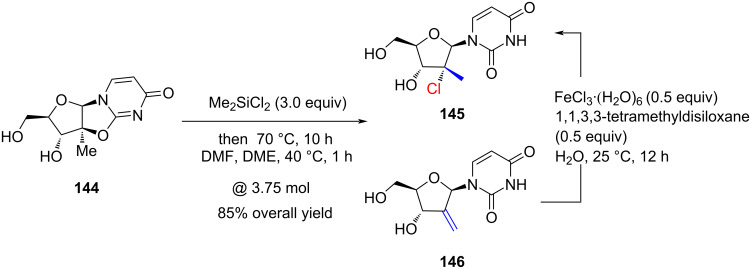
Optimization of the Boger protocol by researchers from Merck [88–89].

Very recently, a modified procedure was reported by researchers from Merck (Scheme 27) [88–89]. They observed that the reaction of **144** with Me_2_SiCl_2_ yielded the desired product **145** along with 5–10% of the undesired elimination byproduct **146**. Subjecting the obtained mixture to the hydrochlorination conditions depicted in Scheme 27 transformed the alkene **146** into chloride **145**. Notably, they observed that no additional chlorine source, such as TsCl, was necessary. Furthermore, they successfully replaced iron oxalate with inexpensive FeCl_3_ hexahydrate and PhSiH_3_ with less costly 1,1,3,3-tetramethyldisiloxane.

### anti-Markovnikov reactions

**Figure 8:**
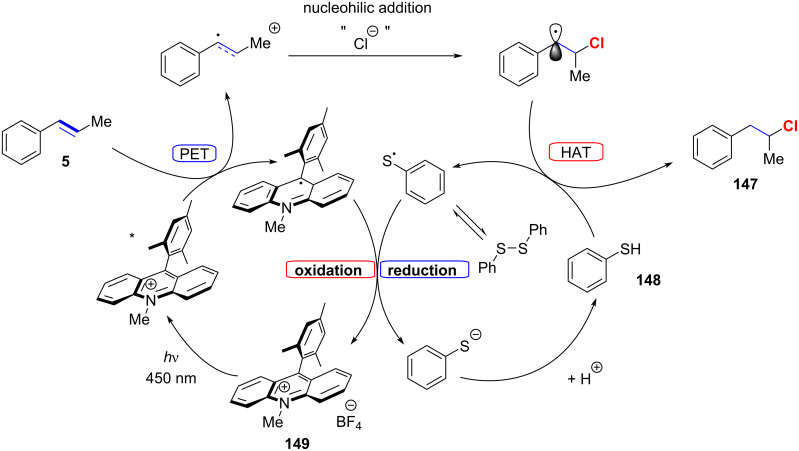
Proposed mechanism for anti-Markovnikov hydrochlorination by Nicewicz.

As stated in the introduction concerning the polar hydrochlorinations the activation energy for an anti-Markovnikov addition is at least by 30 kJ mol^−1^ higher than for normal addition. Therefore, the formation of the anti-Markovnikov product via purely cationic intermediates is never observed. The only report for the formation of the anti-Markovnikov product via polar additions is shown in Scheme 14 (product **79**). In this specific case it was speculated that a competing radical pathway is responsible for the formation of product **79**. Another noteworthy example of an “anti-Markovnikov” addition is shown in Scheme 3. 1-Phenylpropene (**5**) affords what might be called the “Markovnikov product” **6**, whereas dimethylated styrene **1** gives the “anti-Markovnikov” product **2**. Hence, the classification for hydrochlorinations of 1,2-disusbstituted alkenes into Markovnikov and anti-Markovnikov products can be somewhat misleading. The first report which is worthy of being called anti-Markovnikov hydrochlorination was reported by Nicewicz in 2014 [90]. The inversion of regioselectivity is best understood by examination of the proposed catalytic cycle (Figure 8).

First, electronic excitation of photoredox catalyst **149** at 450 nm results in an excited state thereof, denoted with an asterisk, possessing a reduction potential of 2.0 V versus SCE (saturated calomel electrode). Subsequently, this excited state undergoes quenching through photoinduced electron transfer (PET) with styrene **5**. The resulting vinyl radical cation exhibits electrophilicity at the homobenzylic position, engaging in an anti-Markovnikov manner with a formal chloride nucleophile. The ultimate step involves hydrogen atom transfer (HAT) with thiol **148**, culminating in the formation of the desired product **147**.

Therefore, the generation of the vinyl radical cation plays a pivotal role in determining the regioselectivity, with the positive charge being more pronounced on the β-position compared to the α-position. A discussion of the regioselectivity of vinyl cations was already reported in 1973 by Neunteufel and Arnold [91]. They concluded their pioneering paper by stating: “In conclusion we wish to point out that this reaction provides a convenient procedure to achieve anti Markovnikov addition of alcohols to olefins which can presumably be extended to other systems. Furthermore, the addition of other nucleophiles to photochemically generated cation radicals would make this type of reaction of more general synthetic utility”.

**Scheme 28:**
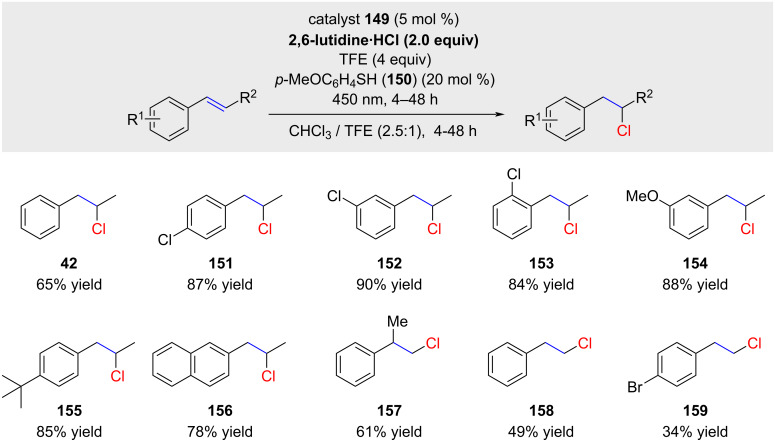
anti-Markovnikov hydrochlorinations as reported by Nicewicz.

Two distinct approaches, denoted as method A and B, were delineated in the study, yielding a relatively restricted scope of products derived from various styrenes bearing few functional groups. The decision to present only the conditions for method B is driven by the fact that, upon comparative evaluation, this method consistently delivers superior results in terms of both regioselectivity and yields for each hydrochlorination product (Scheme 28). Nicewicz and co-workers reported that other electron-rich substrates, such as trialkyl-substituted alkenes, enol ethers, and enamides, proved unproductive in generating the anti-Markovnikov product [90]. The authors attribute this outcome to the high stabilization of the corresponding cations from these substrates, rendering them unresponsive to nucleophilic attack by the chloride anion. Notably, neither the report nor the supporting information provides any indication regarding the reaction temperature (probably room temperature).

**Figure 9:**
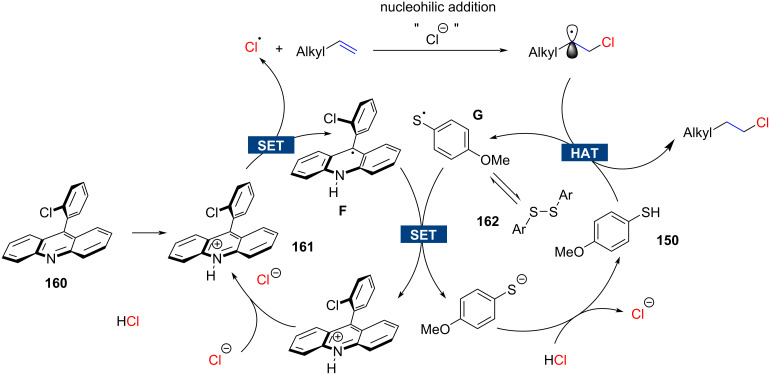
Mechanism for anti-Markovnikov hydrochlorination according to Ritter.

In 2023, Ritter disclosed an anti-Markovnikov hydrochlorination reaction based on hydrochloric acid [92]. Although the proposed catalytic cycle appears to be very similar to the one suggested by Nicewicz [90], the reaction is conceptually distinct (compare Figure 8 and Figure 9). Initially, 9-arylacridine **160**, which is not a photoredox catalyst itself, undergoes protonation by hydrochloric acid to form the corresponding acridinium ion **161**, which in turn is photoredox-active. The acridinium ion **161** now takes on the additional role of a phase-transfer catalyst, facilitating the transport of the chloride ion into the lipophilic alkene phase. Subsequently, under irradiation with blue LEDs, the acridinium cation **161** and the chloride anion engage in a single-electron-transfer (SET) process, generating a chlorine radical and an acridine radical **F**. The chlorine radical adds to the less-substituted terminal position of the alkene to produce the more stable secondary radical. The acridine radical **F** then undergoes a second SET reaction with a thiyl radical **G**, which, upon combination with a chloride anion, regenerates the initial acridinium catalyst **161**. The thiyl radical is formed through hydrogen atom transfer (HAT) with thiol **150**, thus completing the second catalytic cycle. Hence, the key distinction from Nicewicz's work is that in the Ritter protocol, chloride undergoes oxidation, whereas in Nicewicz's report, the alkene is oxidized.

**Scheme 29:**
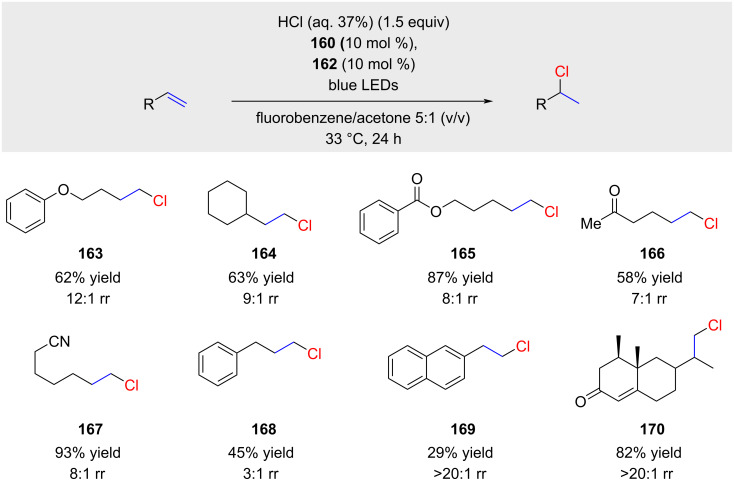
anti-Markovnikov hydrochlorinations as reported by Nicewicz; rr (regioisomeric ratio) corresponds to the ratio of linear (anti-Markovnikov) and branched (Markovnikov) product.

This modification allows for a significantly larger reaction scope (Scheme 29). Terminal alkenes and several functional groups such as ethers (**163**), esters (**165**), ketones (**166**), nitriles (**167**), and enones (**170**) are tolerated. The regioselectivity is in general high but can drop in several cases to relatively low ratios (e,g., product **168**). The reaction works equally well for 1,1-disubstituted alkenes as demonstrated by example **170**. A limitation of the method is its use of hydrochloric acid, making it potentially unsuitable for highly acid-sensitive substrates. Another challenge, shared with Nicewicz's method [90], is the preparation of arylacridine **159** in a single step from the relatively expensive 9-chloroacridine through Pd-catalyzed cross-coupling with 2-chlorophenylboronic acid. Additionally, for large-scale reactions, a flow reactor is necessary [93].

**Scheme 30:**
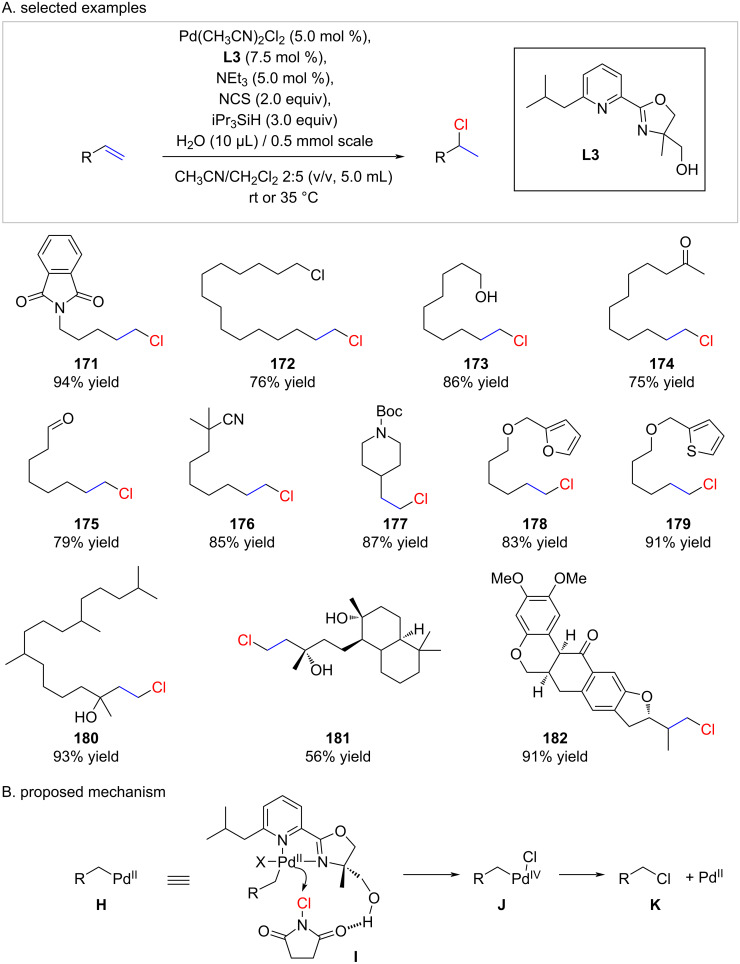
anti-Markovnikov hydrochlorinations as reported by Liu.

In 2022, Liu reported a palladium-catalyzed chain walking–hydrochlorination reaction [94]. While the concept of chain walking is well-established [95], the subsequent reaction of terminal palladium metal with a chlorine electrophile can be considered innovative. This review specifically focuses on the conversion of terminal alkenes into their corresponding chlorides, with the chain-walking aspect not being the primary focus. Hence, we decided to display only the hydrochlorination reactions of “unwalked” terminal alkenes (Scheme 30A).

In contrast, to classical polar hydrochlorinations the functional tolerance of this methodology is striking. Especially examples with sensitive aldehyde (**175**), nitrile (**176**), *N*-Boc (**177**), furan (**178**), thiophene (**179**), and even tertiary alcohols (**180** and **181**) are impressive. The primary drawback of this methodology lies in the synthesis of the ligand **L3**, requiring four steps, coupled with the expense of the palladium catalyst.

Interestingly, when the hydroxy group in **L3** was protected, the chemoselectivity of the reaction was poor, resulting in a 1:2 mixture of the desired chloride and the corresponding terminal alkene. Liu and colleagues put forth the following mechanism (Scheme 30B): Initially, the terminal palladium species **H**, formed through the hydropalladation of terminal or internal alkenes (upon chain walking), coordinates to NCS via hydrogen bonding (**I**). Subsequent oxidation takes place to yield a Pd(IV) species (**J**), which then undergoes reductive elimination, resulting in a Pd(II) complex and the corresponding alkyl chloride **K**.

## Conclusion

Despite being regarded as uninteresting museum chemistry for a considerable time, recent advancements in the hydrochlorination of alkenes have significantly expanded its applicability. Approximately three decades ago, only a few functional groups were tolerated, and the hydrochlorination of terminal, unactivated alkenes was considered very slow or even impossible. However, recent methodologies have overcome these limitations, enabling the hydrochlorination of molecules containing various functional groups. Notably, terminal aliphatic alkenes can now be hydrochlorinated under mild conditions at room temperature. The industrial application of this reaction by Merck underscores its practical utility for pharmaceutical production. While high functional group tolerance is achievable for polar hydrochlorinations with activated alkenes, extending this tolerance to the polar hydrochlorination of terminal alkenes remains a challenge. Metal-catalyzed radical hydrochlorination reactions have emerged as a practical solution, providing a versatile approach to hydrochlorinate a wide range of alkenes. Methods such as ours (Scheme 23), based on acetic acid and hydrochloric acid, as well as Merck's procedure involving inexpensive FeCl_3_ hexahydrate (Scheme 28), facilitate the synthesis of secondary and tertiary chlorides on mole scales. With numerous methodologies now available, the focus shifts to the next question: What applications can be explored for these chlorides? We anticipate that the accessibility of numerous secondary and tertiary chlorides will catalyze substantial research endeavours for the development of innovative reactions with secondary and tertiary chlorides. Lastly, it should be noted that we are not aware of a single report concerning the catalytic asymmetric hydrochlorination of alkenes. Hence, this represents another important challenge for the future.

## Data Availability

Data sharing is not applicable as no new data was generated or analyzed in this study.
